# The rhizosphere microbiome of 51 potato cultivars with diverse plant growth characteristics

**DOI:** 10.1093/femsec/fiae088

**Published:** 2024-06-05

**Authors:** Benoit Renaud Martins, Viviane Radl, Krzysztof Treder, Dorota Michałowska, Karin Pritsch, Michael Schloter

**Affiliations:** Research Unit for Comparative Microbiome Analysis (COMI), Helmholtz Zentrum München, German Research Center for Environmental Health (GmbH), Ingolstädter Landstraße 1, 85764 Neuherberg, Germany; Research Unit for Comparative Microbiome Analysis (COMI), Helmholtz Zentrum München, German Research Center for Environmental Health (GmbH), Ingolstädter Landstraße 1, 85764 Neuherberg, Germany; Plant Breeding and Acclimatization Institute – National Research Institute in Radzików, Bonin Division, Department of Potato Protection and Seed Science at Bonin, Bonin Str 3, 76-009 Bonin, Poland; Plant Breeding and Acclimatization Institute – National Research Institute in Radzików, Bonin Division, Department of Potato Protection and Seed Science at Bonin, Bonin Str 3, 76-009 Bonin, Poland; Research Unit for Environmental Simulation (EUS), Helmholtz Zentrum München, German Research Center for Environmental Health (GmbH), Ingolstädter Landstraße 1, 85764 Neuherberg, Germany; Research Unit for Comparative Microbiome Analysis (COMI), Helmholtz Zentrum München, German Research Center for Environmental Health (GmbH), Ingolstädter Landstraße 1, 85764 Neuherberg, Germany; Chair for Environmental Microbiology, Department of Life Science Systems, School of Life Sciences, Technical University of Munich, Alte Akademie 8, 85354 Freising, Germany

**Keywords:** co-occurrence network, community assembly processes, microbiome, plant growth, potato cultivars, rhizosphere

## Abstract

Rhizosphere microbial communities play a substantial role in plant productivity. We studied the rhizosphere bacteria and fungi of 51 distinct potato cultivars grown under similar greenhouse conditions using a metabarcoding approach. As expected, individual cultivars were the most important determining factor of the rhizosphere microbial composition; however, differences were also obtained when grouping cultivars according to their growth characteristics. We showed that plant growth characteristics were related to deterministic and stochastic assembly processes of bacterial and fungal communities, respectively. The bacterial genera *Arthrobacter* and *Massilia* (known to produce indole acetic acid and siderophores) exhibited greater relative abundance in high- and medium-performing cultivars. Bacterial co-occurrence networks were larger in the rhizosphere of these cultivars and were characterized by a distinctive combination of plant beneficial Proteobacteria and Actinobacteria along with a module of diazotrophs namely *Azospira, Azoarcus*, and *Azohydromonas*. Conversely, the network within low-performing cultivars revealed the lowest nodes, hub taxa, edges density, robustness, and the highest average path length resulting in reduced microbial associations, which may potentially limit their effectiveness in promoting plant growth. Our findings established a clear pattern between plant productivity and the rhizosphere microbiome composition and structure for the investigated potato cultivars, offering insights for future management practices.

## Teaser

The study on 51 potato cultivars revealed a clear pattern between plant productivity and rhizosphere microbial composition, highlighting distinct features in high-performing cultivars conducive to enhanced plant growth.

## Introduction

A projection of the world’s growing population suggests that the demand for food will likely increase by 70% by 2050 (FAO 2019). Potatoes belong to the most important plants to ensure global food security and are in addition used as renewable resources for many parts of industry (Priedniece et al. 2017, Wijesinha-Bettoni and Mouillé 2019, Bakhsh et al. 2020). In 2021, around 373 million tons of potatoes were harvested worldwide (FAOSTAT 2021). However, potatoes are highly susceptible to abiotic and biotic stressors (Levy and Veilleux 2007, Beddington 2010, Monneveux et al. 2013, Bakhsh et al. 2020), and therefore it is assumed that in future yields will be strongly impacted mainly as a result of climate change (Hijmans 2003), which exacerbates prolonged drought periods, increased temperatures, salinity, and emerging pathogens (Handayani et al. 2019, Raza and Bebber 2022). Therefore, there is a strong need to develop alternative forms of management, as traditional agriculture practices will unlikely meet the demand to maintain yields and quality (Singh et al. 2020, Sun et al. 2021). Here, making use of the functional potential of the root-associated microbiome has been proposed as highly promising to ensure global food security while maintaining a healthy environment (Singh and Trivedi 2017).

The rhizosphere is the narrow zone of soil that is influenced by root secretions and drives complex plant–microbe interactions (Berendsen et al. 2012, Beattie 2018). The plant rhizosphere harbors diverse microbes, many of which extend plant capabilities including adaption to environmental stresses (Pascale et al. 2020), resistance against pathogen (Berendsen et al. 2018), and improvement of nutrient and water uptake (Richardson et al. 2009, Haney et al. 2015). While the composition of rhizosphere microbiota is strongly influenced by the soil microbiome (Inceoǧlu et al. 2012, Liu et al. 2019, Veach et al. 2019), in addition plants themselves actively shape microbial assemblages within the rhizosphere through the release of root exudates (Bulgarelli et al. 2013). For potato, those exudates can account for up to 20% of the total assimilated carbon (Gschwendtner et al. 2011), but composition and amount of root exudates can differ among potato cultivars and plant development stages (Gschwendtner et al. 2011). Indeed several studies demonstrated an significant effect of potato cultivars on microbial communities in their rhizospheres (Inceoǧlu et al. 2010, 2011, 2012, Gschwendtner et al. 2011). Copiotrophic bacteria (known as r-strategists), particularly Proteobacteria are enriched in the rhizosphere, when compared to the surrounding soil, as demonstrated in a recent global study that integrated data from various ecosystems and soil backgrounds (Ling et al. 2022). Consequently bacteria genera like *Sphingobium, Bradyrhizobium, Devosia, Microvirga, Rhizobium, Variovorax*, or *Burkholderia*, which have been reported as potentially beneficial for plant growth, have been often described as important bacteria of the potato rhizosphere (Inceoǧlu et al. 2010, Barnett et al. 2015, Pfeiffer et al. 2017). However, besides Proteobacteria, other groups including Acidobacteria, Actinobacteria, Bacteroidetes, Firmicutes, as well as fungi including Ascomycota, Basidiomycota, or Zygomycota are typical member of the microbiome of the potato rhizosphere. Thus assembly processes of the rhizosphere microbiome are essential to understand (Stegen et al. 2013).

Determinism and stochasticity are the two fundamental ecological processes that govern microbial assembly (Vellend 2010, Stegen et al. 2012). While deterministic processes reflect host selection, species filtering by environmental adaptation and interspecies interactions, stochasticity involves trait- and selection-independent community assembly regulated by stochastic events such as random proliferation, death, and dispersal (Stegen et al. 2013, Zhou and Ning 2017). Despite the occurrence of both processes in most communities at local scales (Liu et al. 2023), previous studies reported that the rhizosphere microbiome composition is driven by deterministic processes (Zhang et al. 2018) and is selected according to the functional traits that benefit plant health (Mendes et al. 2014). Recently, another study involving eight potato cultivars grown under continuous cropping regime also demonstrated a prevalent contribution of deterministic processes (64.19%–81.31%) in bacteria community assembly while the fungal communities were mainly dominated by stochastic processes (78.28%–98.99%) (Gu et al. 2022).

Deploying microbial network analyses, especially co-occurrence networks based on DNA sequencing data sets offer great potential for unraveling the hidden patterns within large and complex microbial communities, and therefore have been largely used to study community structures (Barberán et al. 2011, Banerjee et al. 2019, Niraula et al. 2022). These networks are interpreted as reflecting inter- or intrakingdom interactions between species that play diverse roles within the microbial ecosystems (Floc’h et al. 2020, Matchado et al. 2021). Previous studies reported significant differences between microbial co-occurrence networks of bulk soil and those in the rhizosphere (Shi et al. 2016, Ling et al. 2022), mainly attributed to root exudates by which plants selectively recruit specific microbes (Blagodatskaya et al. 2014). Within association networks, nodes with extensive connections were referred to as hub taxa (Agler et al. 2016) and subsequently to keystone taxa, as their removal resulted in outsized impact on both the composition and functioning of the microbiome (Banerjee et al. 2018). Microbial co-occurrence networks can also involve the presence of modules (closely interconnected nodes), which can be interpreted as groups of taxa with overlapping ecological niches (Niraula et al. 2022). A study showed that various cultivars of Rabbiteye Blueberry had a substantial effect on the properties (modularity and robustness) of the rhizosphere bacteria co-occurrence network (Jiang et al. 2017). Furthermore, the authors demonstrated a positive and significant correlation between fruit yield and putative keystone taxa affiliated to Acidobacteria, Proteobacteria, and Actinobacteria (Jiang et al. 2017). In a recent study, members of modules significantly positively correlated with yield were found to be more abundant in the rhizosphere of soybean with high productivity (Niraula et al. 2022). These findings suggest that microbial associations may have implications for plant productivity. Apart from these examples, very little is still known about the relationship between structure and complexity of microbial networks and the growth characteristics of plants, especially in potato.

Although studies have previously reported on the influence of genotype and environmental factors on the potato rhizosphere microbiome (Inceoǧlu et al. 2010, 2011, 2012, Gschwendtner et al. 2011, Weinert et al. 2011, Pfeiffer et al. 2017, Faist et al. 2023), they have only focused on a limited number of potato cultivars, thus providing only a snapshot of the large genetic variability present within this crop. We have expanded the scope by examining a collection of 51 potato cultivars and related them to the rhizosphere microbiome with the aim to: (i) gain a comprehensive understanding of the selective effect potato cultivars have on the composition and structure of the rhizosphere microbiome; (ii) explore microbial co-occurrence patterns in response to plant growth characteristics. Furthermore, we used the β-nearest taxon index (βNTI) approach in a first attempt to understand which assembly processes contribute to the structuring of microbial communities in the rhizosphere of the investigated potato cultivars.

To achieve these goals, we grew the 51 cultivars under controlled greenhouse conditions in soil from an agricultural field to allow for recruitment of a natural rhizosphere microbiome. We hypothesized that under these optimal growth conditions, the rhizosphere microbiome would show differentially higher relative abundances of plant growth-promoting Proteobacteria, including *Microvirga, Variovorax, Hyphomicrobium, Sphingobium*, and *Bradyrhizobium* in high-performing potato cultivars compared to low-performing cultivars (H1). Furthermore, we expected that when the rhizosphere microbiome plays an important role in plant productivity, the microbial co-occurrence network within high-performing cultivars would be larger and more complex than that of low-performing potato cultivars (H2).

## Materials and methods

### Site description and experimental design

The soil for the experiment was collected in summer 2020 from the upper layer (0–20 cm) of an arable field at the experimental station Gut-Roggenstein (latitude 48.1824420, longitude 11.3126896, 508 m above sea level), Technical University of Munich in Southern Germany. The field had previously undergone a series of crop rotations: beans in 2015, wheat in 2016, rapeseed in 2017, wheat in 2018, maize in 2019, and summer barley in 2020. The soil, a loamy sand (55.9% sand, 27% silt, and 17% clay) classified as of luvisol was homogenized with a 2-mm diameter mesh sieve, following the removal of stones and crop residues before the experiment. The soil was stored at 4°C until further use.

The selection of 51 potato cultivars for this study includes varieties intended for food production and industrial purposes, name and characteristics, including country of origin, year of breeding, maturity, skin color, and shape of tubers are provided in Table 1. Further information such as cultivar ID, resistance to abiotic and biotic stresses can be found on the European Cultivated Potato database available at www.europotato.org. These cultivars exhibited significant variability in the quality and quantity of root exudates (Joana Falcao Salles, personal communication), and were chosen specifically to investigate their interaction with the soil microbiome. The cultivars were obtained from the Polish *in vitro* potato gene bank (Bonin, Poland). Plants were cultivated as tissue cultures for ~56 days within the accredited gene bank laboratories at the Bonin Division of the Plant Breeding and Acclimatization Institute—National Research Institute (Bonin, Poland). The Polish Service of Plant Health and Seed Inspection issued a phytosanitary certificate, and disease-free plants were sent to the Helmholtz Munich Center (Munich, Bavaria, Germany). Since the plants were grown in *in vitro* conditions supplemented by Murashige and Skoog nutrient medium, the agar plugs attached to their roots were gently eliminated using tweezers and tap water.

**Table 1.** Name and characteristics of the 51 potato cultivars.

Cultivar	Origin	Year	Earliness	Purpose	The color of the pulp	Skin color	Shape of tuber
ACKERSEGEN	Germany	1929	Late	Table	Yellow	Yellow	Round oval
AMEX	The Netherlands	1965	Medium late	Starch	Yellow	White	Oval
ANIELKA	Poland	1997	Medium late	Table	Creamy	White	Round
APOLLO	Germany	1956	Medium late	General purpose	Yellow	White	Round oval
APTA	Germany	1951	Medium late	General purpose	Pale yellow	Yellow	Oval
ARRAN BANNER	Great Britain	1927	Medium late	Table	Pale yellow	Yellow	Oval
ASTRID	Germany	1969	Medium late	Table	Pale yellow	White	Oval
ATLANTIC	USA	1976	Medium late	Table	White	Yellow	Round
ATOL	Poland	1978	Medium late	Table	Pale yellow	White	Round oval
AULA	Germany	1974	Medium late	Table	Yellow	Yellow	Round oval
BALLADE	The Netherlands	1999	Medium late	Table	Pale yellow	White	Long
BAŁTYK	Poland	1951	Late	Starch	Creamy	Rose	Round oval
BELLADONNA	Germany	1973	Late	Table	Yellow	Beżowy	Round oval
BIHORO	Japan	1969	Very late	General purpose	Yellow	Yellow	Round oval
BODENKRAFT	Germany	1963	Late	Industrial	Pale yellow	White	Round
BRDA STARA	Poland	1964	Late	Table	Yellow	White	Round
CAJKA	Czech Republic	1960	Medium late	Table	Yellow	Yellow	Round oval
CARIBOO	Canada	1968	Medium late	Industrial	White	White	Oval
CARNEA	Germany	1938	Late	Industrial	White	Red	Oval
CERES	Germany	1970	Medium late	Table	Pale yellow	White	Oval
COBRA	Germany	1966	Medium late	Table	Pale yellow	White	Round oval
COSIMA	Germany	1959	Medium late	General purpose	Yellow	White	Round
DANUTA	Germany	2009	Medium late	Starch	Yellow	Yellow	Round oval
DESIREE	The Netherlands	1962	Medium late	Table	Pale yellow	Red	Long oval
DONELLA	Germany	1989	Medium late	Table	White	White	Round oval
ERDKRAFT	Germany	1958	Medium late	Industrial	Creamy	White	Round
FIANNA	The Netherlands	2000	Medium late	Table	White	Yellow	Long oval
HERBSTFREUDE	Germany	1962	Medium late	General purpose	Pale yellow	White	Oval
INWESTOR	Poland	2005	Late	Starch	Creamy	Yellow	Round oval
JANKA	Poland	1976	Late	Table	Creamy	White	Round
JELLY	Germany	2005	Medium late	Table	Yellow	White	Round oval
KAMA	Poland	1978	Medium late	Table	White	White	Oval
KING EDWARD VII	Great Britain	1902	Medium late	Table	Yellow	Yellow	Long oval
KMIEC	Poland	1935	Medium late	General purpose	White	White	Long
KRAB	Poland	1967	Medium late	General purpose	Pale yellow	White	Round
MONI	Germany	1972	Medium late	Industrial	Pale yellow	White	Oval
ORLIK	Czech Republic	1963	Medium late	General purpose	Pale yellow	White	Round
PASJA POMORSKA	Poland	2000	Medium late	Starch	Pale yellow	White	Round oval
RUDAWA	Poland	2002	Late	Starch	Creamy	Yellow	Round oval
SAGITTA	Germany	1961	Late	Table	Yellow	White	Long oval
SALTO	Poland	1998	Medium late	Table	Pale yellow	Yellow	Round oval
SYRENA	Poland	2002	Medium late	Table	Yellow	Yellow	Long oval
SZYPER	Poland	2014	Medium late	Starch	Creamy	White	Round
TEWADI	Germany	1989	Medium late	Table	Yellow	Rose	Round
URSUS	Poland	2004	Late	Table	Creamy	White	Round oval
WIEBKE	Germany	1971	Medium late	Industrial	Yellow	White	Round oval
WOLFRAM	Poland	1999	Medium late	Table	Pale yellow	Yellow	Oval
WYSZOBORSKI	Poland	1955	Late	General purpose	Pale yellow	White	Long oval
ZAGŁOBA	Poland	2007	Late	Table	Yellow	Yellow	Round oval
ZENIA	Poland	2010	Medium late	General purpose	Yellow	Yellow	Round oval

**Figure 1.**
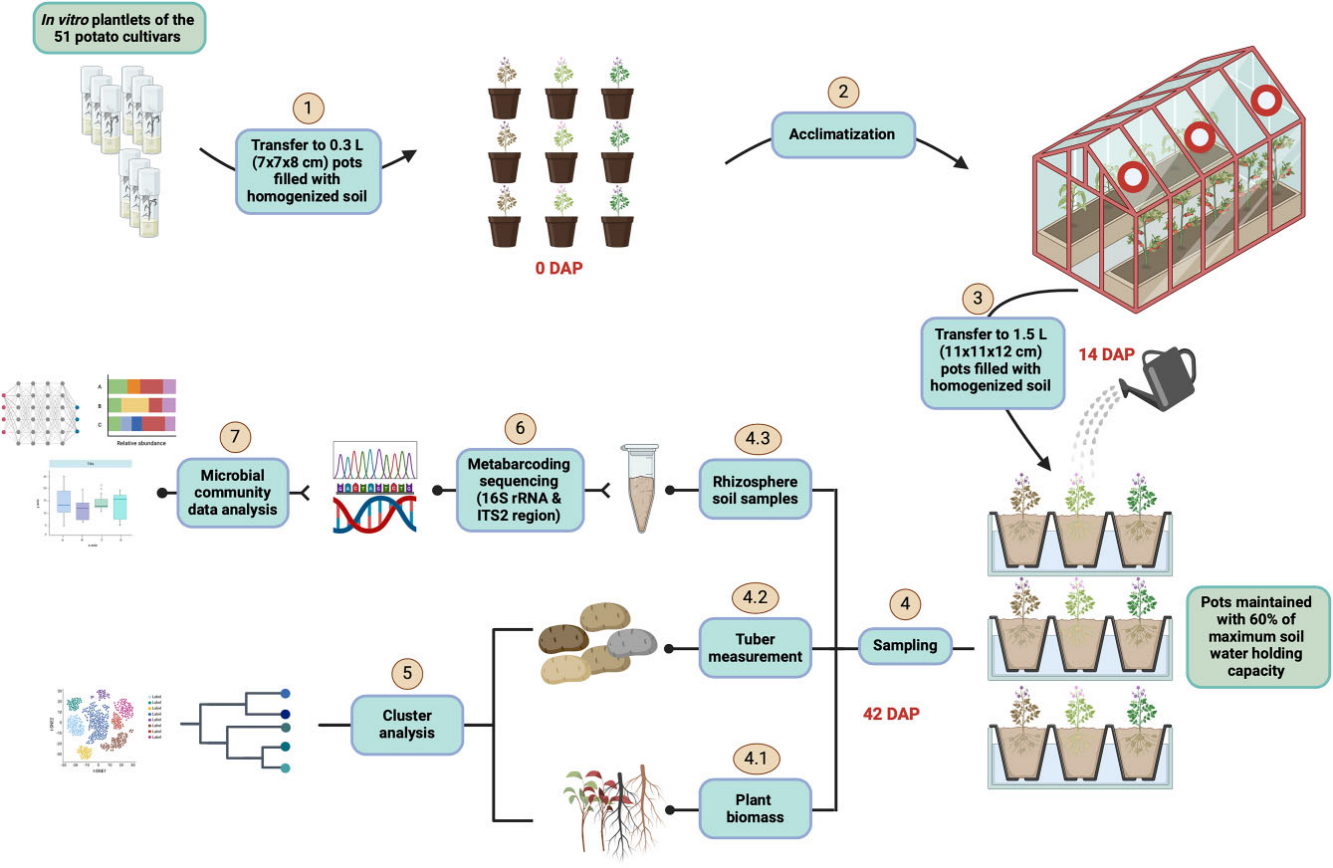
Experimental design of the greenhouse experiment using 51 *in vitro* propagated potato cultivars acclimated in homogenized soil for 14 days. Acclimated plants were transferred to 1.5 l pots containing the same soil and were cultivated for 28 days under continuous watering [60% maximum water holding capacity (mWHC)]. At 42 days after planting, the experiment was terminated. Root, stem, and leaf samples were taken to assess growth traits and rhizosphere soil samples were collected for bacterial and fungal community analyses. Created with BioRender.com.

Each plant was immediately transferred to 0.3 l (7 cm × 7 cm × 8 cm) pots filled with the homogenized soil and allowed to acclimate for 14 days (Fig. 1). Throughout the acclimatization period, the plants were watered three times a week to maintain a roughly 60% of the maximum water holding capacity. Subsequently, the acclimated plants were individually transferred to 1.5 l (11 cm × 11 cm × 12 cm) pots and cultivated for 28 days in the greenhouse under relative humidity of 65%, day/night temperatures of 22°C/18°C, and a day/night photoperiod of 16/8 h. Each pot was regularly weighted, and soil moisture was maintained at 60% of the maximum soil water holding capacity throughout the experiment. Each pot received 50 ml of a low concentration nutrient solution (Table S1, Supporting Information) corresponding to an addition of 1.1 mg N per pot (∼1 kg N ha^−1^) at 16 days after planting in order to not influence soil microbiome with plants. Each cultivar was grown in three replicates. The experiment was terminated 42 days after planting, and various plant growth parameters above- and belowground were recorded (Fig. 1). Shoot fresh weight was measured immediately, and after drying for 2 days at 75°C in the oven, dry weight was promptly recorded. Fresh weight and number of tubers for each individual plant were reported as well as the average number of leaves. These plant growth data are available in Table S2 (Supporting Information).

For each individual plant, the thin layer of soil attached to the plant roots, referred to as the rhizosphere, was collected, resulting in 153 samples in total (51 cultivars × 3 replicates). These samples were immediately placed on dry ice and then stored at –80°C until DNA extraction.

### Plant data analysis

To categorize the 51 potato cultivars according to their growth characteristics, we employed k-means clustering. After calculating the mean values for all growth variables within each cultivar, our data were scaled. To ascertain the optimal number of clusters (k), we employed the R function fviz_nbclust() from the Factoextra package v1.0.7. Visualization of the k-means clusters was accomplished through a principal component analysis using the fviz_cluster() function provided within the same R package. For the reproducibility of the k-means cluster analysis, samples were hierarchically clustered with the hclust() method using Euclidean distance and ward.D2 linkage. Statistical differences between plant growth-based clusters of potato cultivars were assessed with analysis of similarity (ANOSIM) available in the package vegan v2.6–4. The resulting growth clusters (high, medium, and low productivity) were used to analyse the rhizosphere microbial community composition, co-occurrence networks as well as community assembly processes.

### Metabarcoding of bacterial and fungal communities

Total DNA was extracted from 250 mg of each individual rhizosphere soil sample using the DNeasy PowerSoil Kit (QIAGEN GmbH, Hilden, Germany) following the manufacturer’s instructions. Empty extraction tubes were used as negative controls to check for contamination during the process. The concentration of DNA extracts was quantified in duplicate using SpectraMax Gemini EM Microplate Spectrofluorometer (Molecular Devices, CA, USA) and Quant-iT PicoGreen dsDNA Assay Kit (Thermo Fischer Scientific, Waltham, MA, USA) according to the manufacturer’s instructions. All samples were stored at −20°C until further analysis.

The ITSmix3/ITSmix4 primer pair (Tedersoo et al. 2014), was used to amplify the ITS2 region of the fungal nuclear rRNA. PCR was performed with an initial denaturation phase at 95°C for 15 min and 30 cycles of 30 s denaturation at 95°C, 30 s annealing at 55°C and 1 min extension at 72°C, and a final extension of 10 min at 72°C. PCR reaction mixtures contained 1 of 10 ng DNA templates, 0.5 µl of 10 pmol of each primer, 2.5 µl of 3% BSA, 12.5 µl of NEBNext High-Fidelity 2x PCR Master Mix (New England Biolabs, Frankfurt am Main, Germany), and 8 µl of DEPC-treated water, resulting in a total volume of 25 µl.

For amplification of the V4 region of the bacterial 16S rRNA gene, we used universal primer pair 515F/806R (Apprill et al. 2015, Parada et al. 2016). PCR was performed under the following conditions: an initial denaturation phase at 98°C for 1 min and 30 cycles of 10 s denaturation at 98°C, 30 s annealing at 55°C and 30 s extension at 72°C, and a final extension for 5 min at 72°C. PCR reaction mixtures contained 1 µl of 10 ng DNA templates, 0.5 µl of 10 pmol of each primer, 2.5 µl of 3% BSA, 12.5 µl of NEBNext High-Fidelity 2x PCR Master Mix (New England Biolabs), and 8 µl of DEPC-treated water.

PCR products were verified in 1% agarose gels, followed by MagSi NGSprep Plus bead purification (Steinbrenner, Wiesenbach, Germany). The quality and quantity of purified amplicons and the presence of primer dimers were checked with DNF-473 Standard Sensitivity NGS Fragment Kit (1–6000 bp) on a fragment analyser (Agilent Technology, Santa Clara, CA, USA). Both bacterial and fungal purified amplicons were indexed in an 8-cycle PCR, which contained 2.5 µl of each indexing primer (Nextera® XT Index Kit v2; Illumina, San Diego, CA, USA), 12.5 µl NEBNext High-Fidelity 2x PCR Master Mix, 6.5 µl DEPC-treated water, and 10 ng of the purified amplicon. Subsequently, a second round of purification, followed by quality and quantity control were performed as described above. Prior to sequencing, samples were diluted to 4 nM and equimolarly pooled into a single Eppendorf tube. Paired-end sequencing was carried out using the MiSeq^®^ Reagent kit v3 (600 cycles) on the Miseq instrument^®^ (Illumina Inc., San Diego, CA, USA).

Preprocessing of the bacterial and fungal raw sequencing data was conducted on the Galaxy web platform (Afgan et al. 2016) as previously described by Kublik et al. (2022), with few modifications. Briefly, the following trimming and filtering parameters were considered for bacteria: 20 bp were removed n-terminally and reads were truncated at position 220 (forward) and 150 (reverse) with expected error of 3 and 4, respectively. For fungi, forward reads were trimmed to 20–220 bp, reverse reads to 20–160 bp with the same number of errors. After merging reads, the resulting unique amplicon sequence variants (ASVs) were trained against SILVA database v138.1 for bacteria and UNITE fungi database v9.0 released for QIIME, with 0.99 confidence threshold (Abarenkov et al. 2022). The R language and environment v4.2.1 were used for downstream analysis. Using Bioconductor decontam package v1.13.0 (Davis et al. 2018), contaminant sequences were filtered based on prevalence across negative controls, along with ASVs assigned to chloroplast and mitochondria. A phyloseq object was created for each of bacterial and fungal datasets using the Phyloseq package v1.42.0. Singletons (ASVs represented by only one read across all samples) were removed. Furthermore, only ASVs found in at least two out of three replicates per cultivar were kept for downstream analysis. We employed total-sum scaling (TSS) for data normalization. TSS involves transforming the abundance table into a relative abundance table by scaling the data according to the library size of each sample.

## Microbial data analysis and statistics

### Diversity and composition

Alpha diversity within bacterial and fungal communities was estimated through the observed species richness, Shannon index, and Simpson’s dominance index in both, individual cultivars and the growth clusters employing the Microbiome package v1.20.0. A nonparametric Wilcoxon test was conducted to determine the influence of the individual cultivars and plant growth-based clusters on alpha diversity. Difference between sample groups were considered significant when *P* < .05.

Beta diversity was evaluated through a principal coordinates analysis (PCoA) of weighted UniFrac dissimilarity. A permutational multivariate analysis of variance (PERMANOVA) with 999 permutations using weighted UniFrac distance (R package Vegan v2.6–4) was employed to estimate the relative contributions of various factors including individual cultivars, plant growth-based clusters, breeding purpose, earliness and country of origin, on the structure of microbial communities.

Differentially abundant taxa at different taxonomic levels within the plant growth-based clusters of potato cultivars were identified by pairwise Wilcoxon test (*α* = 0.05) adjusted with FDR.

Bacterial and fungal ASVs found in 80% of samples with a relative abundance greater than 0.01% were considered for core microbiome analysis using the amp_venn function available in the ampvis2 package (v2.7.34).

### Microbial assembly process based on entire-community null model

The βNTI and Raup–Crick-based Bray–Curtis (RCbray) were used to determine the contribution of deterministic and stochastic assembly processes as previously described (Stegen et al. 2013). Briefly, the βNTI measures the degree of deviation of the β-mean-nearest taxon distance from the null expectations based on 1000 random shuffles of ASVs across the phylogenetic tree. Values of |βNTI| > 2 indicate deterministic selection, which can be further partitioned into heterogeneous (βNTI > 2) or homogenous (βNTI < 2) selection. While heterogeneous selection implies that selective pressures drive communities to divergent configurations, in homogeneous selection, these selective pressures push communities toward a common composition (Stegen et al. 2015). The remaining community pairs with |βNTI| < 2 indicate that the community is mainly assembled by stochastic processes. RCbray can be used to further classify this stochastic fraction. Values of RCbray < −0.95 indicate communities influenced by homogenizing dispersal (taxonomically more similar than expected; populations are capable of interactions, allowing members to freely exchange), while RCbray > 0.95 suggest dispersal limitation; populations are unable to mix leading to development via ecological drift. Values of |RCbray| < 0.95 indicate an undominated processes, where no single assembly process is capable of explaining variation (Stegen et al. 2015). The βNTI, RCbray, and assembly processes based on entire-community null models (Stegen et al. 2013) were calculated using the qpen function from the iCAMP v1.5.12 R package (Ning et al. 2020).

### Co-occurrence networks

Microbial networks were constructed using the NetCoMi package v1.1.0 (Peschel et al. 2021). Initially, the dataset was subset according to the growth clusters of potato cultivars. After taxa were agglomerated at the genus level (package Speedyseq v0.5.3.9018), the abundance table of each growth cluster underwent center log ratio transformation (package SpiecEasi v1.1.2) to address compositionality bias. To compute associations between taxa, a Pearson correlation with a default threshold of 0.3 was performed. Additionally, sparsification and selection of connected nodes were conducted using a Student *t*-test with α = 0.0001 and 0.05 for bacterial and fungal networks, respectively. False positive was addressed using lFDR < 0.2. The resulting networks were structured into modules using the cluster_fast_greedy method. Nodes possessing the highest eigenvector centrality (above the 95% quantile the empirical distribution of centrality values) were designated as hub taxa. Eigenvector centrality measures the importance of a node within a network based on both its connections (degree) and the centrality of the nodes it is linked to. Lastly, the Fruchterman–Reingold layout algorithm from the package igraph v1.3.5 was used for network visualization.

## Results

As expected, the potato cultivars were the strongest factor shaping the composition of both, the bacterial (PERMANOVA, *R*^2^ = 0.4, *P* = .003) and fungal (PERMANOVA, *R*^2^ = 0.4, *P* = .001) communities in the rhizosphere (Table 2). This was further confirmed with the PCoA of weighted UniFrac distance, which showed a broad and uneven distribution of cultivars along the two axes (Figure S1, Supporting Information). However, the alpha diversity based on observed species richness and Shannon’s index was similar amongst most of these cultivars (Figure S2, Supporting Information). Within rhizosphere bacterial communities, most abundant taxa included Vicinamibacteria (Acidobacteriota), Gammaproteobacteria (Proteobacteria), and Actinobacteria (Actinobacteriota) (Figure S3, Supporting Information). A similar pattern was found in the bare soil prior to planting (Figure S4, Supporting Information). Rhizosphere fungal communities were mainly represented by U. Sordariales, *Ramophialophora, Pseudeurotium, Podospora, Neoschizothecium, Fusarium, Lasiosphaeris*, and *Cercophora* (Figure S5, Supporting Information). In the next step, we were interested in how rhizosphere communities (bacteria and fungi) were associated with growth clusters.

**Table 2.** PERMANOVA testing the relative contribution of different factors on the structure of fungal and bacterial communities in the potato rhizosphere.

	Bacteria			Fungi		
Factor	*R* ^2^	*P*.format	*P*.signif	*R* ^2^	*P*.format	*P*.signif
Cultivars	0.4	.003	**	0.4	.001	***
Growth clusters	0.03	.005	**	0.021	.025	*
Country of origin	0.06	.07	NS	0.039	.689	NS
Breeding year	0.31	.005	**	0.289	.02	*
Breeding purpose	0.03	.038	*	0.033	.006	**
Shape of tubers	0.02	.33	NS	0.038	.05	*
Earliness	0.02	.05	*	0.023	.039	*

*R* ^2^ stands for the variance explained by each factor. NS *P* > .05, *P < .05, **P < .01, and ***P < .001. Analysis was computed with 999 permutations.

### The 51 cultivars can be grouped into plant growth-based performance clusters

**Figure 2.**
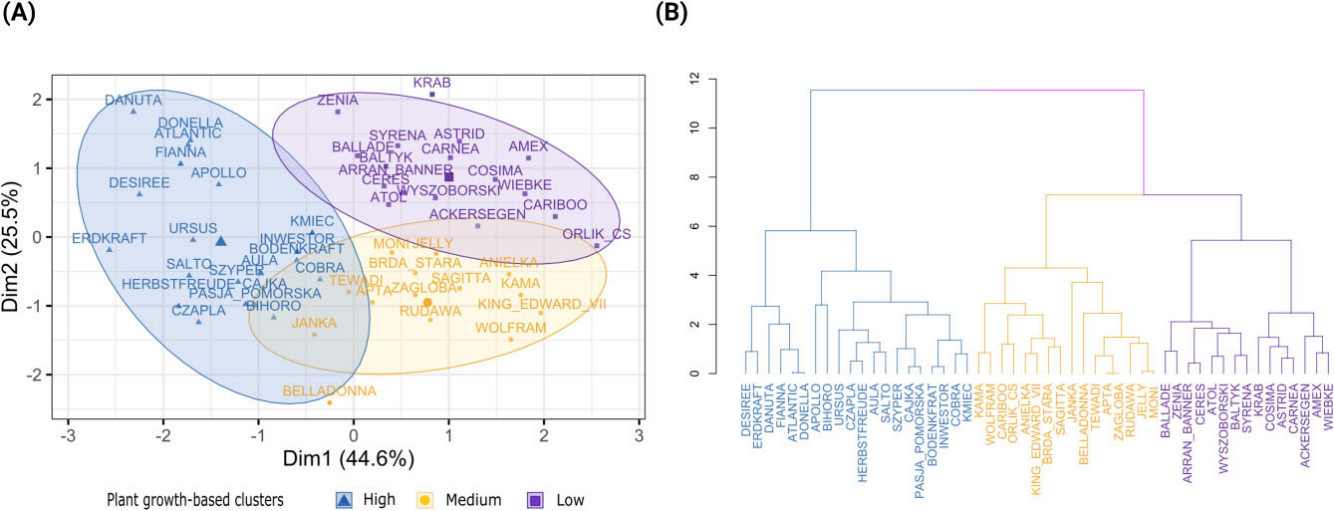
Cluster analysis using (A) k-means and (B) hierarchical clustering. 51 different potato cultivars were grouped in three plant growth-based using shoot biomass, average number of leaves, tuber weight, and numbers upon harvest at 42 days after planting. k-means was performed using the lowest within-sum of squares (means the sum of distances between the points and the corresponding centroids for each cluster) on the Euclidean distance. Hierarchical was performed using ward.D2 linkage on the Euclidean distance.

The 51 potato cultivars grouped into three distinct clusters based on their growth characteristics, and each cluster contained a relatively similar number of cultivars. Both approaches, the k-means (Fig. 2A) and hierarchical clustering (Fig. 2B) resulted in the same number of potato growth clusters with largely identical cultivars. The first cluster comprised high-performing cultivars, the second cluster had cultivars with medium-performance, and the third cluster represented low-performing cultivars. The analysis of similarity between these growth clusters revealed a significant difference (ANOSIM, *R* = 0.6, *P* = .001) among the three groups.

### Plant growth-based clusters influence the diversity and composition of the rhizosphere microbiome

**Figure 3.**
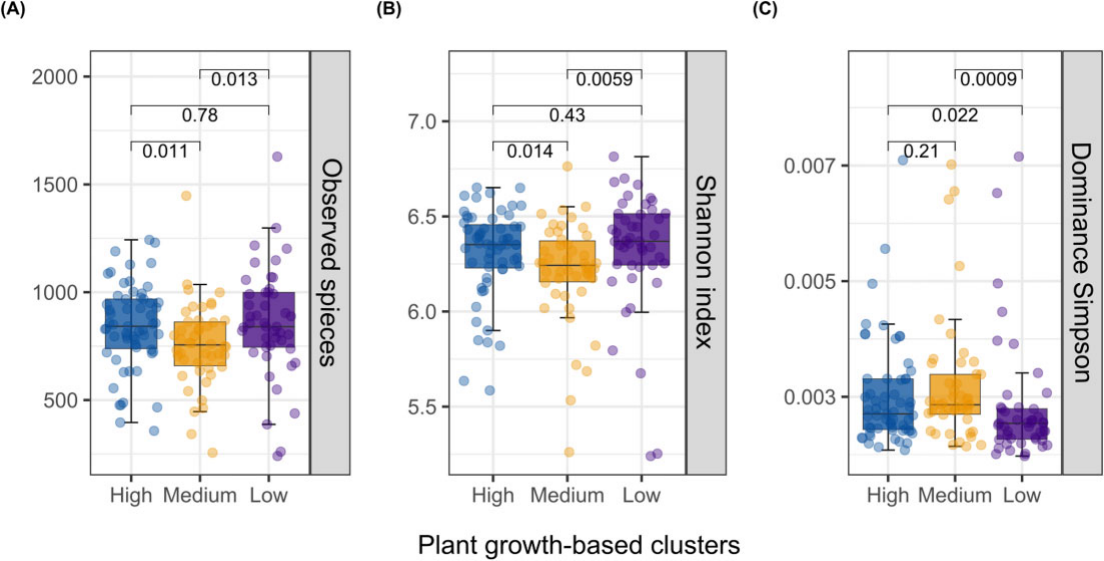
Analysis of bacterial α-diversity. (A) Observed species richness, (B) Shannon's index, and (C) Simpson’s dominance index of the rhizosphere bacterial community in plant growth-based clusters of potato. Boxplots display the medians, tops, and bottoms of the boxes represent 75th and 25th quartiles, and whiskers outside this range; dots illustrate the individual observations in each cluster. Pairwise Wilcoxon test (*P* < .05) was applied to calculate significant differences between potato clusters and numbers above the boxes indicate the corresponding *P*-values of the comparison.

Plant growth-based clusters of potato differed significantly in the bacterial (Figure S3, Supporting Information) but not the fungal α-diversity (Figure S6, Supporting Information). Specifically, the growth clusters comprising high- and low-performing cultivars consistently exhibited a higher observed species richness and Shannon's index compared to that of medium-performing cultivars (Wilcoxon, *P* < .05) (Fig. 3A and B). However, no significant difference in α-diversity was observed between clusters of high- and low-performing cultivars (Wilcoxon, *P* > .05). However, the Simpson's index indicated lower species dominance in low-performing cultivars compared to the other clusters (Wilcoxon, *P* < .05) (Fig. 3C).

**Figure 4.**
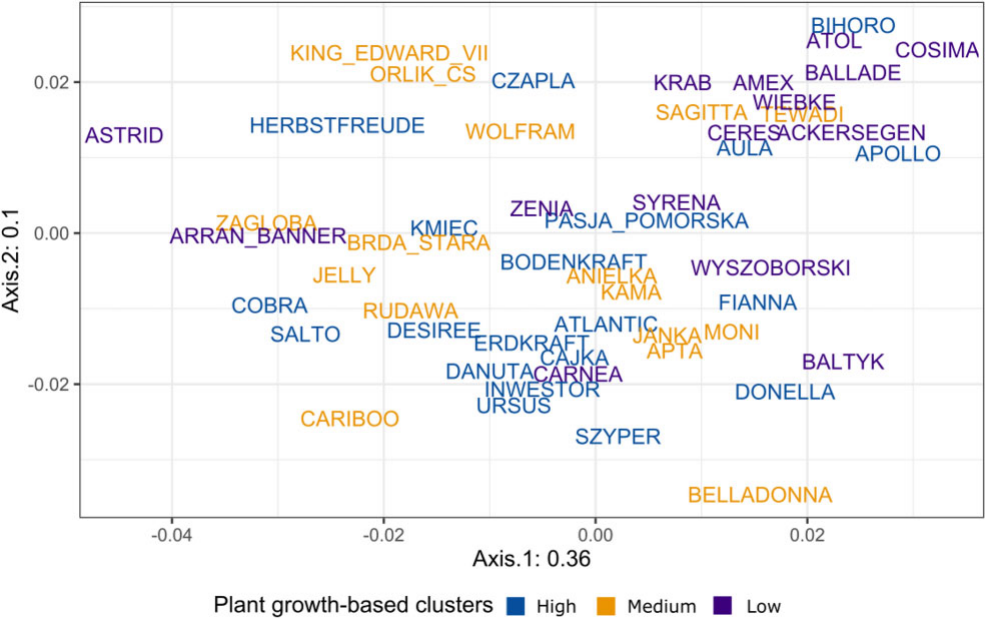
Ordination plot based on PCoA of beta diversity in the rhizosphere fungal communities based on weighted UniFrac distance.

Although overall bacterial and fungal community composition showed no separation according to the plant growth-based clusters of potato (Fig. 4 and Figure S7, Supporting Information), we found that these growth clusters as well as other factors including breeding year, purpose of breeding, and cultivar earliness had a significant effect on the β-diversity (PERMANOVA, *P* < .05) (Table 2). Additionally, the shape of the tubers only affected fungal communities.

**Figure 5.**
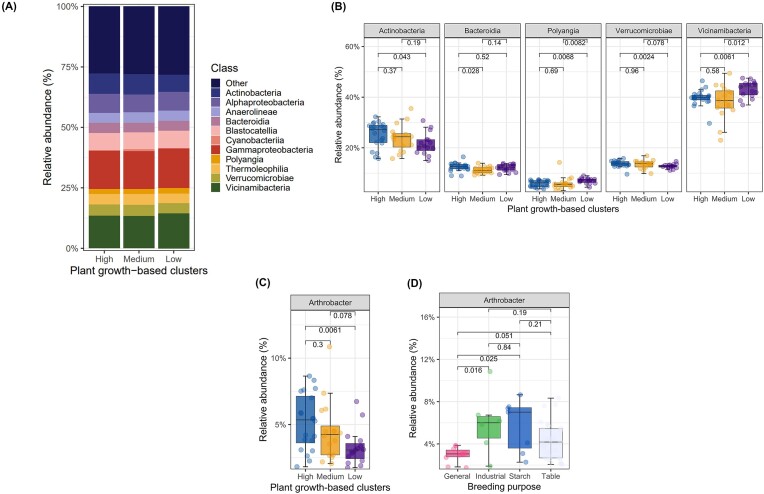
Analysis of bacterial community composition. (A) Relative abundance of the top 10 classes in the rhizosphere of plant growth-based clusters of potato. (B) Pairwise comparison of bacterial classes and (C) bacterial genus *Arthrobacter* between the growth clusters and (D) breeding purpose. Boxplots display the medians, tops, and bottoms of the boxes represent 75th and 25th quartiles, and whiskers outside this range; dots illustrate the individual observations in each cluster. Pairwise Wilcoxon test (*P* < .05) was applied to calculate significant differences across sample groups and numbers above the boxes indicate the corresponding *P*-values of the comparison.

A detailed analysis of microbial community composition of the plant growth-based clusters indicated Vicinamibacteria, Gammaproteobacteria, and Actinobacteria as the three most dominant bacterial classes for all growth-based clusters (Fig. 5A). Pairwise comparisons of the different classes amongst the growth clusters revealed that the relative abundance of Actinobacteria and Verrucomicrobiae was higher in high-performing cultivars in comparison to low-performing cultivars (Wilcoxon, *P* < .05), whereas Vicinamibacteria (Acidobacteriota) and Polyangia (Myxococcota) exhibited the opposite pattern (Wilcoxon, *P* < .05) (Fig. 5B). Due to the low taxonomic resolution, the analysis of Acidobacteriota could only be conducted at the order and family levels. Nevertheless, the same observations were made at both levels, i.e. Vicinamibacterales and Vicinamibacteraceae consistently exhibited higher relative abundance in the cluster of low-performing cultivars. Further analysis at the genus level confirmed the previous results for Actinobacteriota. Specifically, the genus *Arthrobacter*, exhibited higher relative abundance in high-performing cultivars (Wilcoxon, *P* < .05) (Fig. 5C). Additionally, *Arthrobacter* was also influenced by the purpose of breeding as its proportion was found to be greater in cultivars bred for industry and starch production compared to general-purpose cultivars (Wilcoxon, *P* < .05) (Fig. 5D). Neither Alphaproteobacteria nor Gammaproteobacteria showed differential abundances between the growth clusters (Wilcoxon, *P* > .05) (Figure S8, Supporting Information). Genus-based analysis revealed that *Massilia* had a higher proportion in high-performing cultivars compared to low-performing cultivars (Wilcoxon, *P* < .05), MND1 and Ellin6067 showed the opposite trends, and no clear pattern was found for *Sphingomonas* (Figure S9, Supporting Information). Other genera including *Bradyrhizobium, Hyphomicrobium, Lysobacter, Pseudomonas, Thermomonas, Ramibacter, Piscinabcter, Ellin6067, Caenimonas, Acidibacter*, and *Arenimonas* did not discriminate among the growth clusters (Wilcoxon, *P* > .05) (Figure S9, Supporting Information).

The fungal community composition in the rhizosphere of the investigated potato cultivars was dominated by U. Sordariales, *Ramophialophora, Pseudeurotium, Podospora, Neoschizothecium, Fusarium, Lasiosphaeris*, and *Cercophora* (Figure S10, Supporting Information). Amongst these genera, only *Ramophialophora* and *Neoschizothecium* were found to be differentially higher in the cluster of medium-performing cultivars compared to the other two clusters (Wilcoxon, *P* < .05) (Figure S11A, Supporting Information). In addition, *Pseudeurotium* had a higher relative abundance in cultivars used as renewable resources compared to the other cultivars, whereas U. Sordariales showed a lower relative abundance in starch and table potato as compared to the other cultivars (Wilcoxon, *P* < .05) (Figure S11B, Supporting Information).

**Figure 6.**
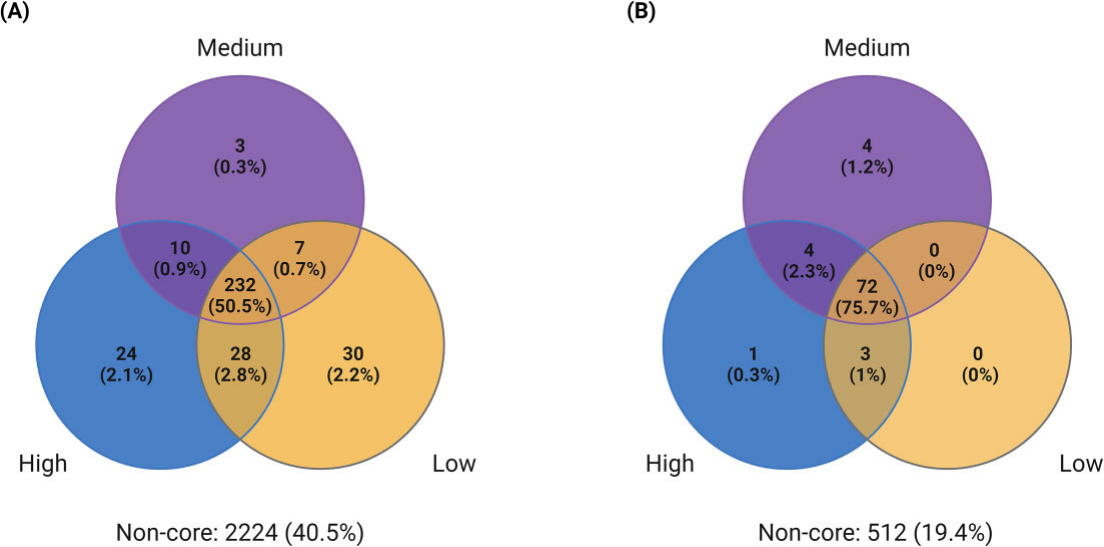
Core microbiome analysis. Venn diagram representing the shared and unique number of (A) bacterial and (B) fungal ASVs in the distinct growth clusters of potato cultivars (colored circles). brackets account for the respective read proportions associated with the ASVs. Core microbiome was defined as taxa consistently found in at least 80% of the samples with minimum relative abundance of 0.01%.

Core microbiome analysis revealed a stable bacterial core microbiome with 232 ASVs (50.5%) as part of the shared community (Fig. 6A). The number of unique ASVs as well as their respective read proportions within each growth clusters was quite small with 24 (2.1%), 3 (0.3%), and 30 (2.2%) unique ASVs in high-, medium-, and low-performing cultivars, respectively. Similar results were obtained for the fungal community where the core microbiome represented 72 ASVs (75.7%) whereby there was marginal or no influence of the growth clusters (Fig. 6B).

### Microbial community assembly and network formation

**Figure 7.**
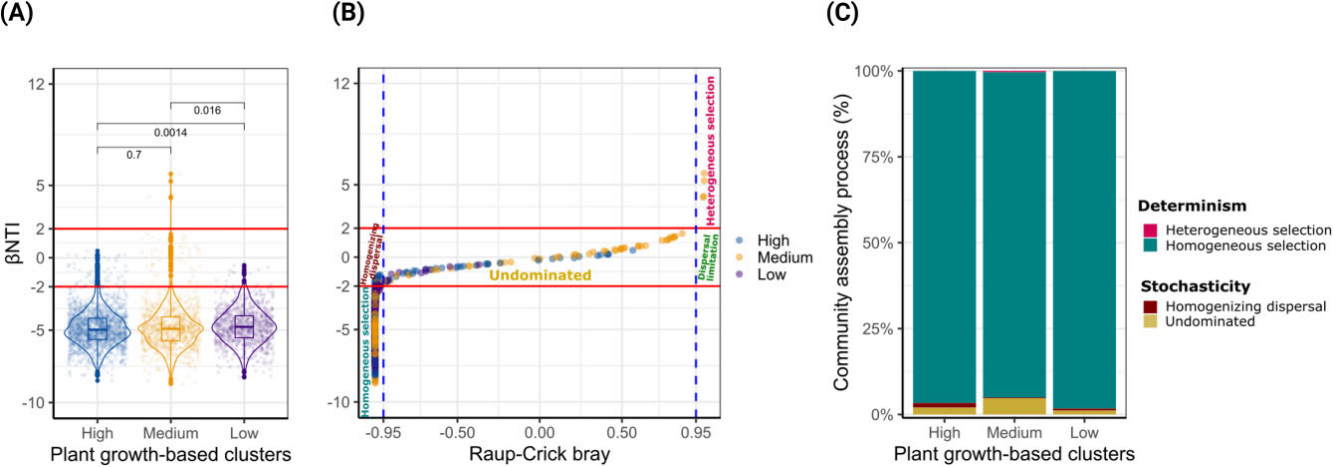
Community assembly processes dominating the rhizosphere bacterial communities in response to the plant growth-based clusters of potato cultivars (A). Differences in βNTI, together with RCbray (B) served to determine the community assembly process that dominated the growth clusters (C). The thresholds for the |βNTI| = 2 and |RCbray| are highlighted as horizontal red lines and the vertical dashed blue lines, respectively. Differences in βNTI are based on Wilcoxon rank-sum test and were considered significant when *P*-value < .05.

Pairwise comparisons of βNTI analysis revealed a significant influence of growth clusters on deviation from the null models, with high- and medium-performing cultivars deviating the most compared to low-performing cultivars (Wilcoxon, *P* < .05) (Fig. 7A). However, across all growth clusters, deterministic processes (|βNTI| > 2) were predominant, primarily characterized by homogeneous selection (βNTI < 2), which contributed to 95%–98% of the assembly of rhizosphere bacterial communities (Fig. 7C). We found heterogeneous selection (βNTI > 2) in medium-performing cultivars, albeit very marginal (Fig. 7C). Among the stochastic processes (|βNTI| < 2), undominated was the most influential pattern observed in the growth clusters, representing a range of 1%–5% (Fig. 7B and C), followed by homogenizing dispersal (up to 1%) in high- and low-performing cultivars (Fig. 7B and C).

By contrast, stochastic processes dominated fungal community assembly, with undominated being the main contributor (75%–80%) followed by homogenizing dispersal (15%–22%) (Figure S12, Supporting Information). Within the deterministic fraction, we found homogeneous and heterogeneous selections, whose contribution ranged between 1% and 10% among the growth clusters (Figure S12, Supporting Information).

**Figure 8.**
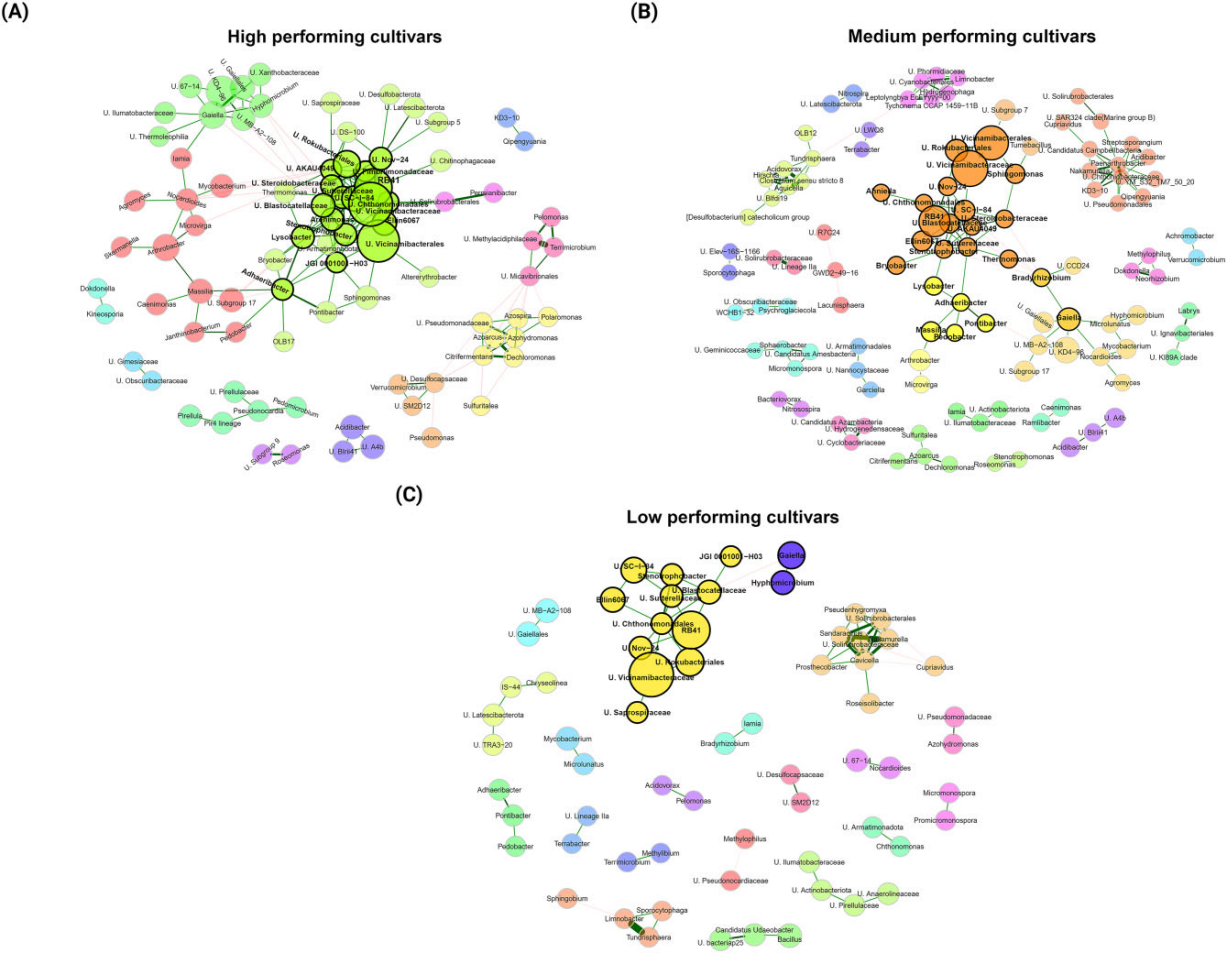
Bacterial co-occurrence network analysis in the rhizosphere according to plant growth-based clusters of potato cultivars. Nodes indicate the genera and edges represent robust (*P* < .0001) Pearson correlation between a pair of nodes. The green and red links represent positive and negative network interactions, respectively. Modules represent group of interconnected nodes, each differently colored. Nodes with thick black borders represent hub taxa, whereas node sizes indicate their respective relative abundances.

The rhizosphere bacterial networks of high- and medium-performing cultivars were larger compared to the cluster of low-performing cultivars (Fig. 8). This difference was evident in the number of connected nodes within the respective networks: 86 nodes in high, 112 in medium, and 65 in low-performing cultivars. Additionally, we found a positive relation between the potato growth clusters and bacterial network complexity, i.e. an increasingly higher network robustness (natural connectivity) and edge density from low- to high-performing cultivars (Table 3). Consequently, the cluster representing low-performing cultivars showed a reduced bacterial co-occurrence network architecture, with the highest average path length observed. Overall, positive edges (Student test, *P* < .0001) represented with green lines were prevalent, ranging from 87.06% to 94.16% (Table 3) in the different networks and seemed to be driven by U. Vicinamibacterales, U. Vicinamibacteraceae, U. Chthonomonadales, U. Rokubacteriales, Stenotrophobacter, U. Blastocatellaceae, and *RB41*. These nodes identified as hub taxa were consistently observed in the different growth clusters with high co-occurrence within the same network modules (nodes of the same color). Moreover, we observed that hub modules were larger in high- and medium- compared to low-performing cultivars. However, medium- and low-performing cultivars exhibited a topological structure with higher modularity and a greater number of modules compared to high-performing cultivars (Table 3). Interestingly, compared with high-performing cultivars, the number of modules containing less than four nodes was 3-fold higher in medium- and low-performing cultivars. Within the network of high-performing cultivars, numerous Actinobacteriota lineages, including *Lamia, Mycobacterium, Gaiella, Nocardioides, Agromyces*, U. 67–14, U. MB-A2-108, *Arthrobacter*, U. Thermoleophilia, and *Pseudonocardia* were mainly distributed across three modules. Moreover, these taxa co-occurred within their respective modules, but also exhibited positive interactions with some plant growth-promoting Proteobacteria, such as *Massilia, Hyphomicrobium*, and *Microvirga*. These observations were overall consistent within the network of medium-performing cultivars. Conversely, for low-performing cultivars, the identified patterns were dispersed in the network and occurred within smaller-sized modules. Lastly, the differentially abundant genera including *Arthrobacter* and *Massilia* were not observed in the network of low-performing potato cultivars.

**Table 3.** Topological features of bacterial co-occurrence network analysis in the rhizosphere according to plant growth-based clusters of potato cultivars. Fungal network properties in the rhizosphere of plant growth-based clusters of potato cultivars. Network property includes number of components, clustering coefficient, modularity, positive edge percentage, edge density, robustness, average path length, and number of modules.

Network properties	High	Medium	Low
Number of components	386	370	422
Clustering coefficient	0.59	0.56	0.55
Modularity	0.51	0.81	0.77
Positive edge (%)	87.06	94.16	89.19
Edge density	0.0019	0.0015	0.0007
Robustness	0.0042	0.0029	0.0025
Average path length	1.41	1.01	1.52
Number of modules	13	24	20
Hub taxa	*Adhaeribacter*, *Arenimonas*, *Bryobacter*, *Ellin6067*, *JGI 0001001-H03*, *Lysobacter*, *RB41*, *Stenotrophobacter*, *Thermomonas*, U. AKAU4049, U. Armatimonadota, U. Blastocatellaceae, U. Chitinophagaceae, U. Chthonomonadales, DS-100, Firmbriimonadaceae, U. Rokubacteriales, U. SC-I-84, U. Solirubrobacteraceae, U. Steroidobacteraceae, U. Sutterellaceae, U. Vicinamibacteraceae, U. Vicinamibacterales, U. Nov-24	*Adhaeribacter*, *Ahniella*, *Bradyrhizobium*, *Bryobacter*, *Ellin6067*, *Gaiella*, *Lysobacter*, *Massilia*, *Pedobacter*, *Pontibacter*, *RB41*, *Sphingomonas*, *Stenotrophobacter*, *Thermomonas*, *U*. AKAU4049, U. Blastocatellaceae, U.Chthonomonadales, U.Rokubacteriales, U.Steroidobacteraceae, U. Sutterellaceae, U. Vicinamibacteraceae, U. Vicinamibacterales, U. Nov-24	*Ellin6067*, *Gaiella*, *Hyphomicrobium*, *JGI 0001001-H03*, *RB41*, *Stenotrophobacter*, U. Blastocatellaceae, U. Chthonomonadales, U.Rokubacteriales, U.Saprospiraceae, U. Sutterellaceae, U. Vicinamibacteraceae, U. Nov-24

Networks of fungal communities were also distinct between the three growth clusters (Figure S13, Supporting Information). Notably, the number of nodes, modules, edge density, and the robustness decreased from high to low-performing cultivars (Table S3, Supporting Information). Hub taxa were most common within high- and medium-preforming cultivars, and included *Dichotomopilus, Dendryphion, Emericellopsis, Thielavia, Gibellulopsis*, and U. Chaetomiaceae, whereas hub taxa of low-performing cultivars were *Myrmecridium, Penicillium, Pseudeurotium, Pseudogymnoascus, Trichoderma, Scutellinia*, and U. Pyronemataceaeae (Figure S13 and Table S3, Supporting Information). Interestingly, the co-occurrence patterns within the hub modules were stronger in high- and medium- than in low-performing cultivars (Figure S13, Supporting Information).

## Discussion

In this study, we assessed the community structure of rhizosphere bacteria and fungi of 51 distinct potato cultivars grown in the same soil under well-standardized conditions in the greenhouse. Furthermore, we investigated microbial association networks and the ecological processes driving microbial community assembly in respect to the plant growth characteristics to understand how plant productivity governs the structure of these microbial communities.

Overall, the observed bacterial and fungal ASVs richness were mostly similar between the distinct potato cultivars used in this study. Moreover, the shared ASVs between all cultivars represented those with the highest read counts, i.e. around 50% and 75% in bacteria and fungi, respectively. This suggests that (i) a significant proportion of the potato microbiome regardless of genotypic differences is necessary for the plant when grown in the same soil, and (ii) this proportion may be variable from one community to another. Nevertheless, individual cultivars had the strongest influence on both bacterial and fungal community composition in the rhizosphere of potato, which is in line with previous reports where significant cultivar or genotype effects in the potato rhizosphere were shown (Gschwendtner et al. 2011, Inceoǧlu et al. 2012, Faist et al. 2023).

Compared with previous research involving a small number of distinct varieties, our approach with the 51 cultivars demonstrated that a significant part of the microbiome was largely conserved in the potato rhizosphere regardless of the differences highlighted above between these genotypes. Using 51 cultivars, we were also able to define different classes of potato on the basis of plant growth parameters, which so far has not been possible with a limited number of cultivars. To our knowledge, this is the first study linking the plant growth characteristics of various cultivars to microbiome characteristics, in order to enhancing our understanding of the role of the rhizosphere microbiome in potato productivity.

### Microbial taxa driving plant performance in the potato rhizosphere

It is worth noting that the statement on plant productivity (low, medium, and high) made for the studied cultivars is specific to this study, and therefore is not indented to be generalized, as these cultivars may perform differently when grown in other soil and climatic conditions. Nevertheless, when they were grouped into plant growth-based clusters, we found a significant impact on rhizosphere microbial communities. Specifically, high- and medium-performing cultivars were dominated by certain bacterial ASVs whereas in the low-performing cultivars, ASVs were more evenly represented amongst samples. Mainly a higher relative abundance of the genera *Arthrobacter* (Actinobacteriota) and *Massilia* (Proteobacteria) were observed in high- and medium-performing cultivars compared to low-performing cultivars. Previously, production of siderophores and phytohormones such as indole acetic acid (IAA) were reported for *Arthrobacter* (Banerjee et al. 2010, Boukhatem et al. 2022), suggesting this genus encompasses a potential plant growth promoters. Another study evidenced an *Arthrobacter* strain to effectively colonize rice roots through biofilm formation, promoting lateral root growth and carrying the potential of antimicrobial activities towards plant pathogens (Chhetri et al. 2022). The genus *Massilia* is a major rhizosphere and plant root-colonizing bacterium, capable to produce IAA, siderophores, and lytic enzymes involved in the control of phytopathogens (Adrangi et al. 2010, Hrynkiewicz et al. 2010, Kuffner et al. 2010, Weinert et al. 2010, Ofek et al. 2012). Taken together, these data suggest that there may be a link between plant growth and specific genera involved in several ways how to promote plant growth. Proteobacteria suggested as plant growth promoters including *Bradyrhizobium, Microvirga, Variovorax, Hyphomicrobium, Sphingomonas*, and *Pseudomonas* were not differentially abundant in the different growth clusters in our study. However, we found the genus *Massilia*, which has similar plant growth promoting traits as the aforementioned genera, in higher relative abundance in the rhizosphere of high-performing cultivars suggesting support for H1 albeit for a different genus than expected.

### Proteobacteria and Actinobacteriota synergy in the co-occurrence network of high-performing potato cultivars

Additionally, we demonstrated that the rhizosphere bacterial communities were dominated by deterministic assembly processes, mainly homogeneous selection (95%–98%) in the different growth clusters. Our findings are consistent with results from other studies where similar observations were reported for potato (Gu et al. 2022), but also for other plant species including soybean (Mendes et al. 2014, Zhang et al. 2018). This indicates that the consistent higher relative abundance of certain beneficial bacteria taxa in the rhizosphere of high- and medium-performing cultivars and no dominance of these species in low-performing cultivars is the result of the homogeneous selection within each growth cluster.

Interestingly, some of the Proteobacteria members suggested as plant growth promoters (*Hyphomicrobium, Microvirga*, and *Bradyrhizobium*), the abovementioned genus *Massilia* as well as other Proteobacteria including *Caenimonas, Skermanella, Janthinobacterium*, and *Pedomicrobium* co-occurred with many Actinobacteriota lineages in high- and medium-performing cultivars. For e.g. *Microvirga*, previously observed in potato rhizosphere (Pfeiffer et al. 2017) are known as nitrogen-fixing and plant growth-promoting bacteria (Ardley et al. 2012), while the genus *Hyphomicrobium* was associated with the suppression of *Ralstonia solanacearum* in tomato-cultivated soil microbiome (Wei et al. 2019). On the other hand, nodes belonging to Actinobacteriota present within these occurrence patterns included *Arthrobacter, Agromyces, Pseudonocardia, Mycobacterium, Iamia, Nocardioides, Gaiella*, and so on. The last two were found in the rhizosphere of Solanaceae, namely tobacco, tomato, and potato cultivars (Hu et al. 2020, Chen et al. 2022, Martins et al. 2023), and endosphere of maize and rice (Kämpfer et al. 2016, Wang et al. 2021), indicating their effective interactions with different plant species. These genera encompass species involved in N fixation and nitrate reduction (Tóth et al. 2008, Albuquerque et al. 2011, Wang et al. 2016, Nafis et al. 2019). These observations indicate that although most of beneficial Proteobacteria genera did not exhibit greater relative abundance in high-performing cultivars, they potentially play a significant role in the recruitment of other beneficial bacteria, thus promoting the growth of these specific cultivars. This was further illustrated by having *Arthrobacter* and *Massilia*, the two differentially abundant genera interconnected, and this pattern was only observed within the network of high- and medium-performing cultivars. Further evidence for the significance of Proteobacteria is the exclusive presence of a module of diazotrophs (biological nitrogen-fixation microorganisms) namely *Azohydromonas, Azospira, Azoarcus*, and *Dechloromonas* (Hurek et al. 2002, Reinhold-Hurek and Hurek 2006, Rodrigues Coelho et al. 2008, Cheng et al. 2023, Guo et al. 2023) in the co-occurrence network of high-performing cultivars and to a lesser extent in medium-performing cultivars. In a previous study, it was shown that reduced NPK inputs stimulated N-fixing activity and the number of diazotrophs in the potato rhizosphere (Volkogon et al. 2021). The presence of diazotrophic genera within the network of high-performance cultivars suggests they may start having a growing influence in the network due to a potential depletion of nitrogen by the plant. It is noteworthy to mention that plants received ~1 kg ha^−1^ of N, which is far lower than the current field applications (180–252 kg N ha^−1^). Conversely, we found fewer and sparse co-occurrence patterns between these beneficial members affiliated to Proteobacteria and Actinobacteriota within low-performing cultivars. This may indicate a less efficient network given that in a previous work, taxa positively correlated with yield were more abundant in the rhizosphere co-occurrence network of soybean with high productivity (Niraula et al. 2022). Additionally, the lowest number of nodes, edge density, robustness, and particularly the highest average path length observed within this growth cluster suggest a network with limited effectiveness. The modularity was ≥ 0.4 implying that all networks exhibited a modular topology (Newman 2006). However, modules with less than four nodes in the network of low-performing cultivars was three times more important compared to those observed in high-performing cultivars. One may speculate that this could have resulted in less efficient plant growth promotion. Bacterial communities being primarily governed by deterministic processes in low-performing cultivars, this implies that the reduced co-occurrence network architecture observed in the latter did not arise by chance, but rather was influenced by the cultivars themselves, since they exert the strongest influence on their rhizosphere microbiome. Overall, we found support for H2, which predicted a larger co-occurrence network with more intricate interactions within high- compared to low-performing cultivars of potato.

Further support for H2 is the largest number of hub taxa and their respective interactions found in the network of high-performing cultivars. However, despite the influence of growth clusters on these taxa, specific nodes in the hub modules primarily affiliated to Acidobacteriota (U. Vicinamibacterace, U. Blastocatellaceae, *Stenotrophobacter*, and *RB41*) emerged as core hub taxa, i.e. they were consistently present within the distinct networks. This persistence suggests a fundamental and stable role of these Acidobacteriota-affiliated nodes in the structure of bacterial communities across diverse contexts, particularly because these taxa, which are highly connected to others do not only frequently act as gatekeepers influencing ecosystem functions (Jiang et al. 2017), but also their removal resulted in outsized impact on both the composition and functioning of the microbiome (Banerjee et al. 2018). Interestingly, previous studies have reported on other Acidobacteria members as key players within co-occurrence networks in soil and plant-associated microbial communities (Banerjee et al. 2016a,b, Jiang et al. 2017). In our study, Acidobacteriota was the most abundant phylum in the potato rhizosphere (U. Vicinamibacteraceae and *RB41* showed the largest node sizes, indicating their prevalence). These results are not aligned with previous studies, which described the rhizosphere as an environment rich in carbon sources, thus favoring the proliferation of copiotrophs, particularly Proteobacteria and Bacteroidetes (Ling et al. 2022). Similarly, the analysis of the bare soil prior to planting revealed the same pattern observed in the rhizosphere, i.e. a prevalence of Acidobacteriota, suggesting that soil microbial background is the primary contributor to the rhizosphere microbiome (Inceoǧlu et al. 2012, Liu et al. 2019, Veach et al. 2019).

Fungal communities were also driven by individual cultivars and the influence of growth clusters as observed in bacteria networks were consistent with fungal network analysis. Overall, we found that fungal communities were assembled by stochastic events, mainly undominated (75%–80%) in the potato rhizosphere. In a recent study (Gu et al. 2022), the analysis of fungal communities in (bulk) soil used for different potato cultivars demonstrated similar patterns (78.28%–98.99%), suggesting that stochastic events are prevalent in fungal assembly irrespective of the compartment (bulk soil and rhizosphere). On the other hand, the difference between rhizosphere microbial communities has been related to the smaller body size of bacteria, their faster growth and higher dispersal rates than fungi, potentially resulting in relatively stronger deterministic processes for colonizing and establishing in new habitats (Powell et al. 2015, Aslani et al. 2022). In contrast, species with a low dispersal rate may be limited in their capacity to colonize different environmental niches, thus experiencing a lower influence of environmental selection on community assembly (Leibold et al. 2004). Therefore, fungi likely underwent stochastic selection patterns (Nemergut et al. 2013, Zinger et al. 2019).

In conclusion, the growth characteristics of potato cultivars drive a deterministic and stochastic assembly of rhizosphere bacterial and fungal communities, respectively. Their influence was further exemplified in microbial co-occurrence patterns, which showed larger networks with more intricate interactions within high-performing cultivars than in low-performing representatives. Within these networks, we found that high-performing cultivars featured unique positive interactions between plant growth-promoting Proteobacteria and Actinobacteriota, mainly *Massilia* and *Arthrobacter* as well as a module of diazotrophs whereas in low-performing cultivars, none of these features were observed. This study demonstrates that distinct cultivars with similar growth characteristics consistently recruit and structure their microbiome according to the patterns described above. We also pinpointed microbial candidates that govern plant growth. Future research work aimed at mapping root exudate patterns of these cultivars with the rhizosphere microbiome activity may provide a better understanding of how plant–microbe interactions can be used to improve crop productivity. It is worth noting that our study involved a single timepoint sampling, thus providing a first insight into the assembly processes of microbial communities in a large number of potato varieties. Further studies including multiple sampling timepoints are required to comprehensively address forces that govern microbial community assembly over a growing season and their implication for potato yield.

## Supplementary Material

fiae088_Supplemental_File

## Data Availability

The sequencing datasets supporting the conclusions of this article are available in the NCBI repository, in the BioProject PRJNA978426.
