# Supplementary material for: The rhizosphere microbiome of 51 potato cultivars with diverse plant growth characteristics
Source: FEMS Microbiol Ecol. 2024 Jun 5;100(8):fiae088. doi: 10.1093/femsec/fiae088 (PMC11242454; doi:10.1093/femsec/fiae088)
Supplement: fiae088_Supplemental_File [file fiae088_supplemental_file.docx]

# Supporting Information

**Table S1.** Modified Hoagland’s nutrient solution (Taiz and Zeiger 2010).

|  | Stock solution (g L^-1^) | 1X (in 1 L) |
| --- | --- | --- |
| Macronutrients |  |  |
| KNO_3_ | 303.30 | 2 mL |
| Ca(NO_3_)_2-_4H_2_O | 472.32 | 2 mL |
| NH_4_H_2_PO_4_ | 230.06 | 1 mL |
| MgSO_4_-7H_2_O | 246.48 | 1 mL |
| Micronutrients |  |  |
| KCl | 3.728 | 1 mL |
| H_3_BO_3_ | 1.546 |  |
| MnSO_4-_H_2_O | 0.338 |  |
| ZnSO_4-_7H_2_O | 0.576 |  |
| CuSO_4_-5H_2_O | 0.124 |  |
| (NH_4_)_6_Mo_7_O_24-_4H_2_O | 0.084 |  |
| Iron ammonium citrate | 5 | 1 mL |

**Table S2.** Plant growth parameters at 42 days after planting, with FW = fresh weight, DW = Dry weight and AVG_LEAVES = Average number of leaves. All weights were recorded in gramme (g).

| Cultivars | Shoot_FW | Shoot_DW | Tuber_number | Tuber_weight | Avg_leaves |
| --- | --- | --- | --- | --- | --- |
| ACKERSEGEN | 3.93 | 0.52 | 0 | 0 | 8 |
| ACKERSEGEN | 4.57 | 0.6 | 3 | 1 | 8 |
| ACKERSEGEN | 4.19 | 0.33 | 0 | 0 | 8 |
| AMEX | 0.23 | 0.03 | 1 | 0.36 | 6 |
| AMEX | 0.16 | 0.02 | 1 | 0.3 | 6 |
| AMEX | 0.16 | 0.02 | 1 | 0.17 | 6 |
| ANIELKA | 1.53 | 0.18 | 2 | 0.43 | 8 |
| ANIELKA | 0.62 | 0.09 | 1 | 0.33 | 8 |
| ANIELKA | 2.77 | 0.35 | 1 | 0.26 | 8 |
| APOLLO | 4.57 | 0.42 | 7 | 3.99 | 8 |
| APOLLO | 4.84 | 0.62 | 3 | 1.55 | 8 |
| APOLLO | 3.25 | 0.24 | 1 | 0.12 | 8 |
| APTA | 4.54 | 0.58 | 3 | 2.23 | 8 |
| APTA | 5.04 | 0.74 | 2 | 2.66 | 8 |
| APTA | 4.6 | 0.68 | 2 | 1.79 | 8 |
| ARRAN_BANNER | 5.36 | 0.71 | 3 | 2.14 | 6 |
| ARRAN_BANNER | 5.23 | 0.74 | 2 | 2.95 | 6 |
| ARRAN_BANNER | 4.86 | 0.64 | 1 | 1.2 | 6 |
| ASTRID | 4.48 | 0.55 | 2 | 1.84 | 6 |
| ASTRID | 4.75 | 0.58 | 2 | 0.3 | 6 |
| ASTRID | 4.12 | 0.48 | 1 | 0.18 | 6 |
| ATLANTIC | 5.44 | 0.7 | 5 | 3.6 | 6 |
| ATLANTIC | 6.02 | 0.69 | 4 | 3.27 | 6 |
| ATLANTIC | 5.52 | 0.6 | 3 | 3.18 | 6 |
| ATOL | 3.89 | 0.59 | 3 | 1.56 | 6 |
| ATOL | 4.64 | 0.63 | 8 | 2.31 | 6 |
| ATOL | 3.26 | 0.46 | 0 | 0 | 6 |
| AULA | 3.78 | 0.5 | 5 | 2.69 | 8 |
| AULA | 3.5 | 0.5 | 3 | 1.15 | 8 |
| AULA | 3.49 | 0.45 | 5 | 2.56 | 8 |
| BALLADE | 4.19 | 0.49 | 3 | 2.24 | 6 |
| BALLADE | 4.28 | 0.57 | 5 | 1.51 | 6 |
| BALLADE | 4.61 | 0.59 | 2 | 0.2 | 6 |
| BALTYK | 3.95 | 0.57 | 2 | 1.46 | 5 |
| BALTYK | 4.56 | 0.67 | 6 | 2.93 | 5 |
| BALTYK | 4.07 | 0.6 | 2 | 1.44 | 5 |
| BELLADONNA | 5.09 | 0.75 | 2 | 1.07 | 10 |
| BELLADONNA | 4.81 | 0.69 | 5 | 3.48 | 10 |
| BELLADONNA | 4.77 | 0.7 | 1 | 2.78 | 10 |
| BIHORO | 1.94 | 0.2 | 1 | 1.89 | 10 |
| BIHORO | 2.53 | 0.29 | 1 | 4.07 | 10 |
| BIHORO | 2.52 | 0.3 | 2 | 3.03 | 10 |
| BODENKRAFT | 3.58 | 0.51 | 4 | 2.72 | 8 |
| BODENKRAFT | 4.04 | 0.47 | 3 | 2.66 | 8 |
| BODENKRAFT | 4.15 | 0.48 | 2 | 1.58 | 8 |
| BRDA_STARA | 5.15 | 0.71 | 2 | 2.6 | 8 |
| BRDA_STARA | 5.53 | 0.78 | 2 | 2.03 | 8 |
| BRDA_STARA | 4.93 | 0.61 | 1 | 0.36 | 8 |
| CAJKA | 5.02 | 0.65 | 4 | 2.54 | 9 |
| CAJKA | 4.74 | 0.6 | 4 | 2 | 9 |
| CAJKA | 4.94 | 0.59 | 4 | 1.98 | 9 |
| CARIBOO | 1.66 | 0.21 | 1 | 0.45 | 6 |
| CARIBOO | 0.6 | 0.09 | 1 | 0.76 | 6 |
| CARIBOO | 0.92 | 0.15 | 1 | 1.09 | 6 |
| CARNEA | 5.15 | 0.71 | 1 | 2 | 6 |
| CARNEA | 5.03 | 0.61 | 3 | 2 | 6 |
| CARNEA | 4.9 | 0.52 | 0 | 0 | 6 |
| CERES | 4.1 | 0.52 | 2 | 1.94 | 6 |
| CERES | 5.15 | 0.73 | 3 | 0.65 | 6 |
| CERES | 5 | 0.68 | 2 | 2.98 | 6 |
| COBRA | 4.31 | 0.63 | 2 | 2.14 | 8 |
| COBRA | 5.14 | 0.59 | 3 | 2.89 | 8 |
| COBRA | 4.53 | 0.62 | 2 | 3.19 | 8 |
| COSIMA | 4.55 | 0.54 | 1 | 0.25 | 6 |
| COSIMA | 3.72 | 0.53 | 2 | 0.46 | 6 |
| COSIMA | 3.23 | 0.44 | 2 | 1.4 | 6 |
| CZAPLA | 3.4 | 0.44 | 4 | 3.42 | 9 |
| CZAPLA | 3.07 | 0.38 | 1 | 4.05 | 9 |
| CZAPLA | 3.68 | 0.56 | 8 | 1.44 | 9 |
| DANUTA | 5.52 | 0.66 | 5 | 3.06 | 5 |
| DANUTA | 4.98 | 0.65 | 6 | 2.94 | 5 |
| DANUTA | 4.1 | 0.51 | 6 | 4.17 | 5 |
| DESIREE | 4.57 | 0.53 | 4 | 3.6 | 7 |
| DESIREE | 3.74 | 0.41 | 4 | 4.17 | 7 |
| DESIREE | 4.27 | 0.5 | 3 | 4.97 | 7 |
| DONELLA | 4.03 | 0.48 | 2 | 2.9 | 6 |
| DONELLA | 4.97 | 0.58 | 5 | 3.8 | 6 |
| DONELLA | 4.79 | 0.55 | 5 | 3.22 | 6 |
| ERDKRAFT | 5.07 | 0.58 | 3 | 4.81 | 8 |
| ERDKRAFT | 4.9 | 0.64 | 3 | 4.65 | 8 |
| ERDKRAFT | 5.43 | 0.64 | 5 | 4.62 | 8 |
| FIANNA | 4.64 | 0.58 | 3 | 3.67 | 6 |
| FIANNA | 4.62 | 0.62 | 3 | 3.83 | 6 |
| FIANNA | 5.35 | 0.66 | 5 | 4.55 | 6 |
| HERBSTFREUDE | 4.84 | 0.86 | 1 | 3 | 9 |
| HERBSTFREUDE | 4.99 | 0.56 | 8 | 3.11 | 9 |
| HERBSTFREUDE | 4.64 | 0.44 | 4 | 3.01 | 9 |
| INWESTOR | 5 | 0.62 | 5 | 2.48 | 8 |
| INWESTOR | 4.42 | 0.54 | 2 | 2.07 | 8 |
| INWESTOR | 4.28 | 0.57 | 2 | 1.98 | 8 |
| JANKA | 4.93 | 0.88 | 2 | 1.19 | 8 |
| JANKA | 4.96 | 0.77 | 1 | 1 | 8 |
| JANKA | 4.61 | 0.64 | 13 | 2.21 | 8 |
| JELLY | 3.72 | 0.57 | 3 | 1.52 | 7 |
| JELLY | 4.63 | 0.64 | 1 | 0.03 | 7 |
| JELLY | 3.51 | 0.49 | 2 | 3.38 | 7 |
| KAMA | 4.07 | 0.62 | 2 | 0.91 | 7 |
| KAMA | 4.03 | 0.63 | 1 | 0.29 | 7 |
| KAMA | 4.04 | 0.68 | 2 | 2.25 | 7 |
| KING_EDWARD_VII | 4.46 | 0.54 | 0 | 0 | 8 |
| KING_EDWARD_VII | 3.84 | 0.68 | 3 | 1.36 | 8 |
| KING_EDWARD_VII | 3.11 | 0.39 | 0 | 0 | 8 |
| KMIEC | 4.66 | 0.6 | 2 | 2.63 | 7 |
| KMIEC | 4.23 | 0.59 | 5 | 2.64 | 7 |
| KMIEC | 4.73 | 0.63 | 1 | 3.13 | 7 |
| KRAB | 3.57 | 0.4 | 3 | 0.64 | 5 |
| KRAB | 3.91 | 0.47 | 4 | 1.63 | 5 |
| KRAB | 3.81 | 0.47 | 0 | 0 | 5 |
| MONI | 4.53 | 0.66 | 1 | 1.27 | 7 |
| MONI | 4.3 | 0.62 | 2 | 1.58 | 7 |
| MONI | 3.86 | 0.53 | 3 | 2.38 | 7 |
| ORLIK_CS | 3.53 | 0.54 | 1 | 0.4 | 7 |
| ORLIK_CS | 4.69 | 0.57 | 0 | 0 | 7 |
| ORLIK_CS | 3.7 | 0.57 | 0 | 0 | 7 |
| PASJA_POMORSKA | 4.61 | 0.57 | 5 | 2.84 | 9 |
| PASJA_POMORSKA | 4.97 | 0.65 | 3 | 1.61 | 9 |
| PASJA_POMORSKA | 5.54 | 0.7 | 3 | 2.4 | 9 |
| RUDAWA | 5.1 | 0.71 | 1 | 1.98 | 8 |
| RUDAWA | 4.88 | 0.72 | 1 | 2.56 | 8 |
| RUDAWA | 4.79 | 0.71 | 2 | 2.11 | 8 |
| SAGITTA | 3.74 | 0.48 | 4 | 1.63 | 8 |
| SAGITTA | 3.61 | 0.54 | 3 | 0.72 | 8 |
| SAGITTA | 2.42 | 0.35 | 0 | 0 | 8 |
| SALTO | 4.53 | 0.61 | 5 | 3 | 8 |
| SALTO | 4.44 | 0.63 | 5 | 3.02 | 8 |
| SALTO | 4.47 | 0.59 | 4 | 3.16 | 8 |
| SYRENA | 3.92 | 0.53 | 3 | 2.46 | 5 |
| SYRENA | 3.42 | 0.5 | 3 | 1.53 | 5 |
| SYRENA | 4.15 | 0.56 | 2 | 2.06 | 5 |
| SZYPER | 4.5 | 0.51 | 5 | 2.12 | 9 |
| SZYPER | 4.39 | 0.57 | 3 | 2.5 | 9 |
| SZYPER | 4.52 | 0.5 | 2 | 2.7 | 9 |
| TEWADI | 4.67 | 0.62 | 4 | 1.4 | 8 |
| TEWADI | 4.65 | 0.66 | 4 | 2.15 | 8 |
| TEWADI | 4.46 | 0.68 | 1 | 2.38 | 8 |
| URSUS | 4.13 | 0.54 | 3 | 3.67 | 7 |
| URSUS | 4.14 | 0.59 | 6 | 3.76 | 7 |
| URSUS | 4.19 | 0.6 | 4 | 3.37 | 7 |
| WIEBKE | 1.22 | 0.14 | 0 | 0 | 7 |
| WIEBKE | 2.44 | 0.3 | 1 | 0.77 | 7 |
| WIEBKE | 1.35 | 0.17 | 1 | 0.05 | 7 |
| WOLFRAM | 4.84 | 0.86 | 4 | 2.16 | 8 |
| WOLFRAM | 3.03 | 0.53 | 2 | 0.37 | 8 |
| WOLFRAM | 3.83 | 0.5 | 0 | 0 | 8 |
| WYSZOBORSKI | 5.21 | 0.76 | 4 | 1.32 | 6 |
| WYSZOBORSKI | 4.35 | 0.55 | 3 | 0.7 | 6 |
| WYSZOBORSKI | 4.16 | 0.58 | 2 | 0.54 | 6 |
| ZAGLOBA | 3.76 | 0.48 | 3 | 3.49 | 8 |
| ZAGLOBA | 4.17 | 0.63 | 2 | 2.29 | 8 |
| ZAGLOBA | 4.22 | 0.64 | 2 | 0.89 | 8 |
| ZENIA | 3.66 | 0.44 | 2 | 1.25 | 5 |
| ZENIA | 4.19 | 0.56 | 2 | 2.4 | 5 |
| ZENIA | 3.76 | 0.47 | 6 | 1.8 | 5 |

## Pre-processing of sequencing data

Following sequencing, one rhizosphere sample replicate from each of the cultivars APTA and FIANNA for 16S rRNA gene library, and from KRAB for ITS library was excluded from the analysis due to low read counts.

**Table S3.** Fungal network properties in the rhizosphere of plant growth-based clusters of potato cultivars.

| **Network properties** | **High** | **Medium** | **Low** |
| --- | --- | --- | --- |
| Nr of components | 89 | 106 | 108 |
| Nr of nodes | 46 | 33 | 28 |
| Clustering coefficient | 0.59 | 0.78 | 0.50 |
| Modularity | 0.48 | 0.38 | 0.65 |
| Positive edge (%) | 81.70 | 82.14 | 80.00 |
| Edge density | 0.0098 | 0.0067 | 0.0036 |
| Robustness | 0.014 | 0.013 | 0.0088 |
| Average path length | 2.45 | 1.31 | 1.80 |
| Number of modules | 11 | 10 | 8 |
| Hub taxa | *Dichotomopilus, Dendryphion, Emericellopsis, Thielavia,* U. Chaetomiaceae, *Periconia, Gibellulopsis* | *Dichotomopilus, Dendryphion, Emericellopsis, Gibellulopsis, Stachybotrys, Thielavia,* U. Chaetomiaceae | *Myrmecridium, Penicillium, Pseudeurotium, Pseudogymnoascus, Trichoderma, Scutellinia*  U. Pyronemataceaeae |


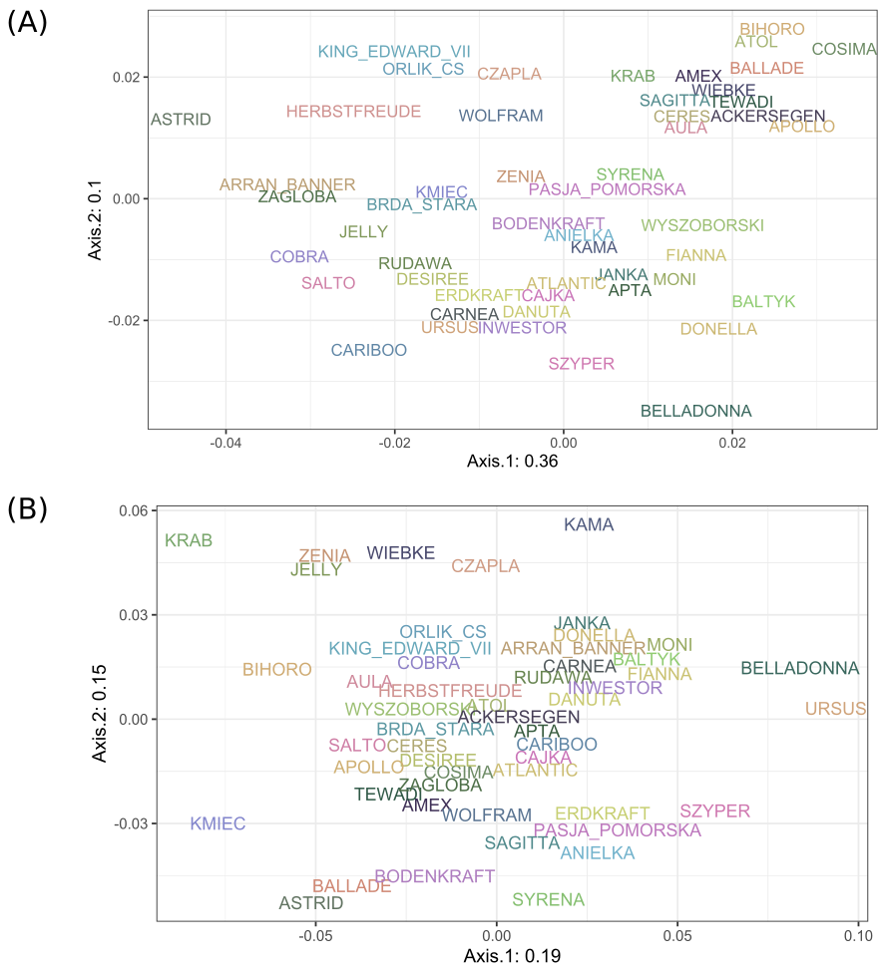


**Figure S1**. Ordination plots based on principal coordinate analysis (PCoA) of beta diversity in the rhizosphere (A) bacterial and (B) fungal communities based on weighted UniFrac distance. Colors indicate the individual potato cultivars.


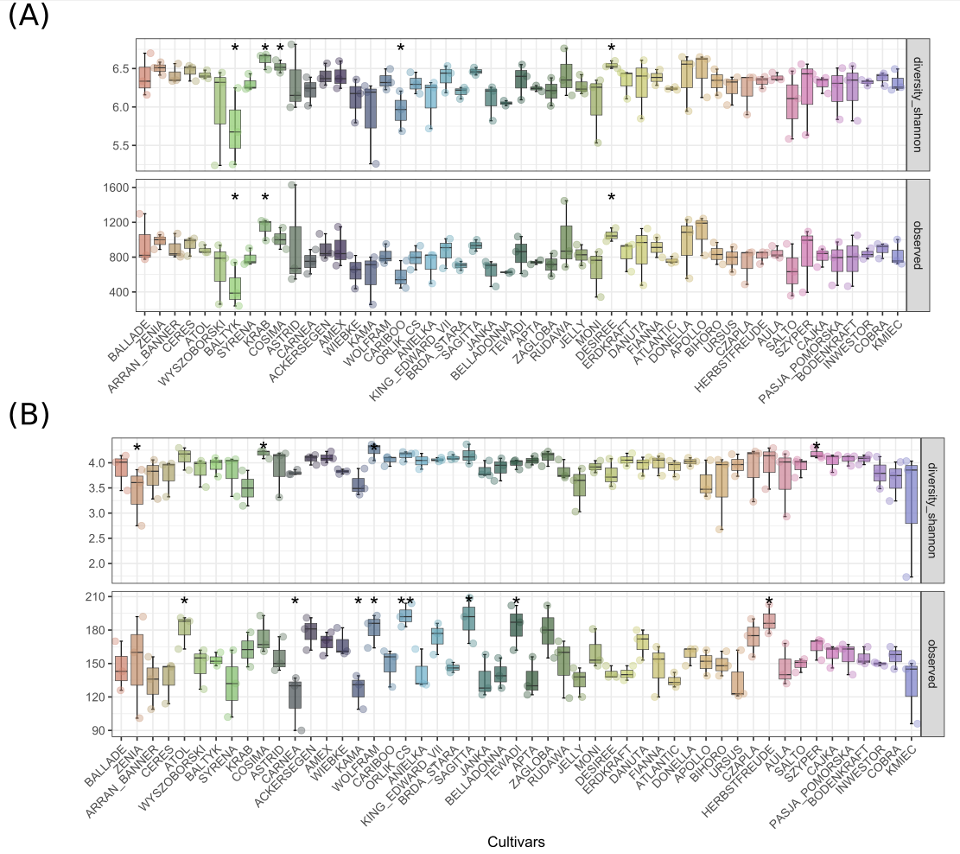


**Figure S2**. Analysis of microbial α-diversity. (A) Bacterial and (B) fungal diversity based on Shannon index and observed species richness in the rhizosphere of 51 distinct potato cultivars. Boxplots display the medians, tops and bottoms of the boxes represent 75th and 25th quartiles, respectively, and whiskers indicate the outsides of this range; dots illustrate the individual observations in each cluster. Colors indicate the different potato cultivars. Pairwise Wilcoxon test (*P* < 0.05) was applied to calculate significant differences between potato clusters and numbers above the boxes indicate the corresponding *P*-values of the comparison.


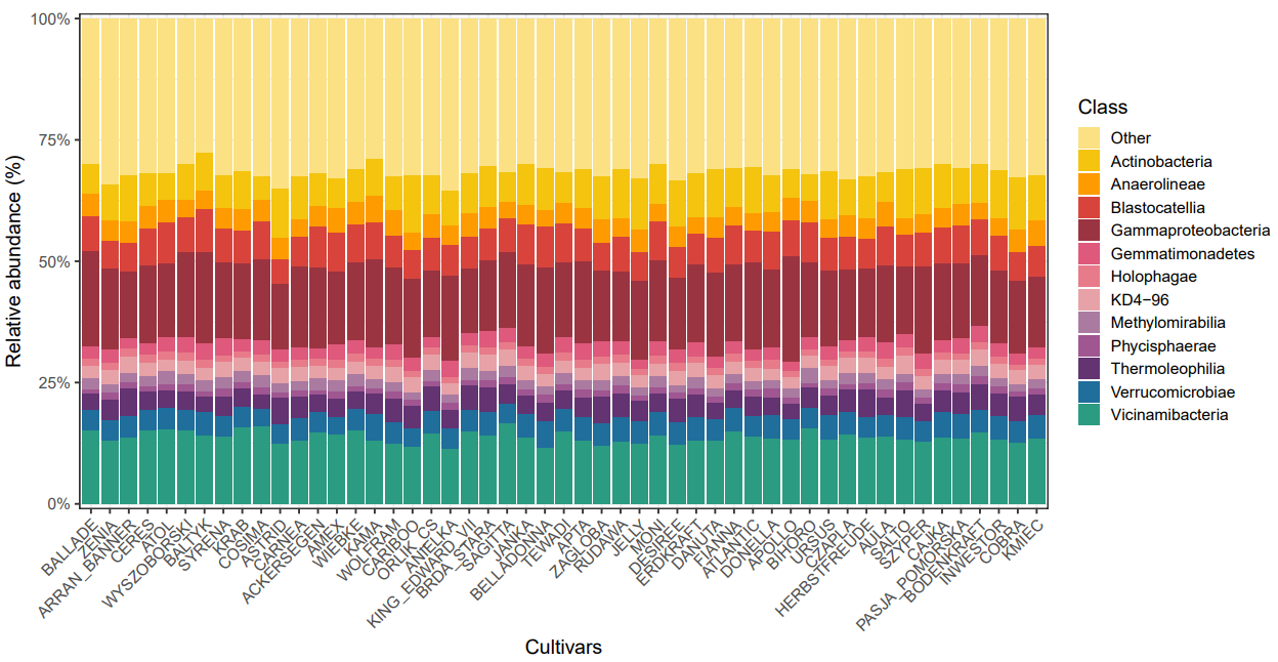


**Figure S3**. Analysis of bacterial community composition. Relative abundance of the top 12 classes in the rhizosphere of the 51 potato cultivars. Colors represent the individual taxa.


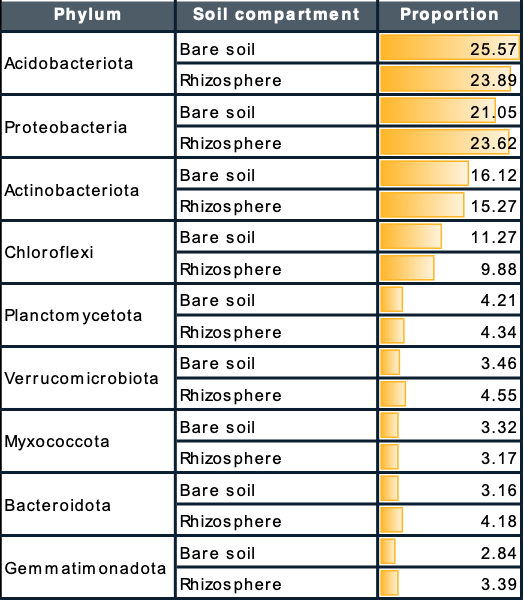


**Figure S4**. Analysis of bacterial community composition. Relative abundance of the top 9 phyla in two soil compartments (bare soil before planting and in the rhizosphere) of the 51 potato cultivars.


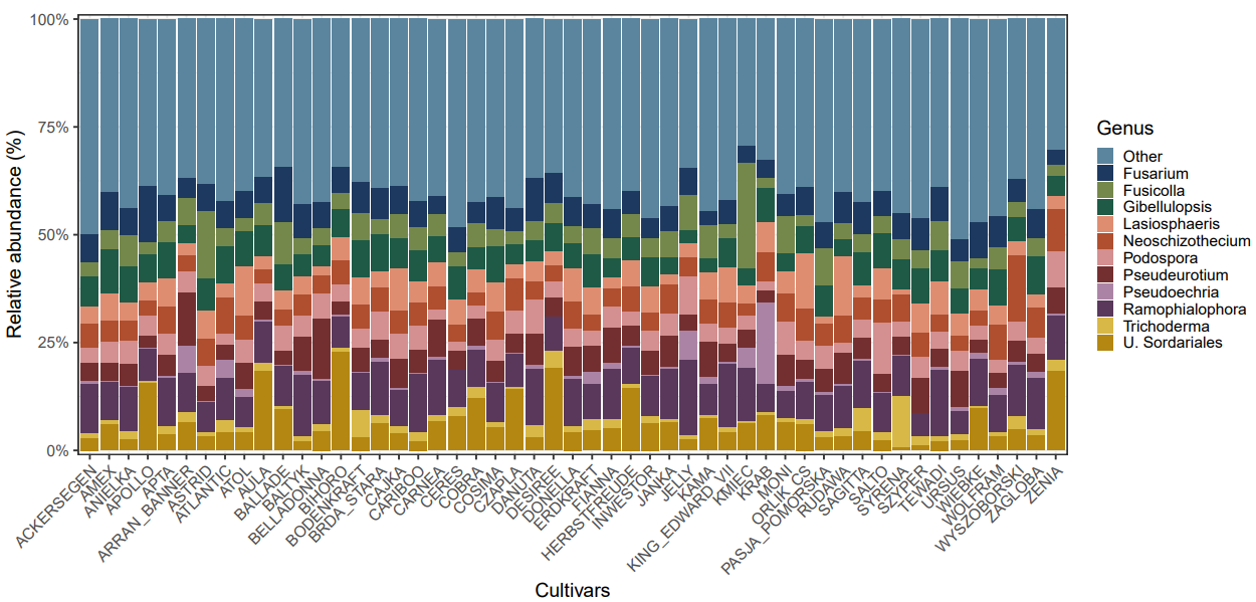


**Figure S5**. Analysis of fungal community composition. Relative abundance of the top 11 genera in the rhizosphere of the 51 potato cultivars. Colors represent the individual taxa.


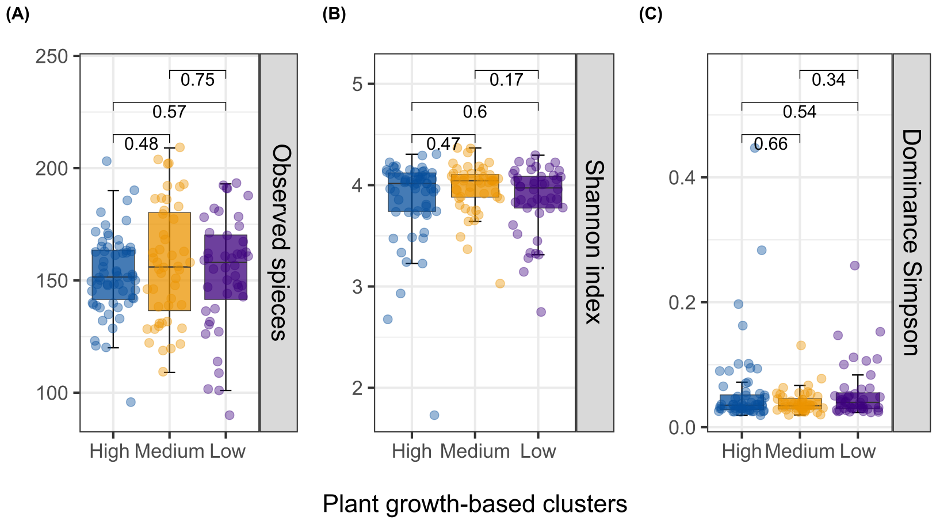


**Figure S6**. Analysis of fungal α-diversity. (A) Observed species richness (B) Shannon index and (C) Simpson’s dominance index of the rhizosphere fungal community in plant growth-based clusters of potato cultivars. Boxplots display the medians, tops and bottoms of the boxes represent 75th and 25th quartiles, and whiskers the outsides of this range; dots illustrate the individual observations in each cluster. Color indicates the different potato clusters (blue: high performing, orange: medium performing, purple: low performing). Pairwise Wilcoxon test (*P* < 0.05) was applied to calculate significant differences between growth clusters and numbers above the boxes indicate the corresponding *P*-values of the comparison.


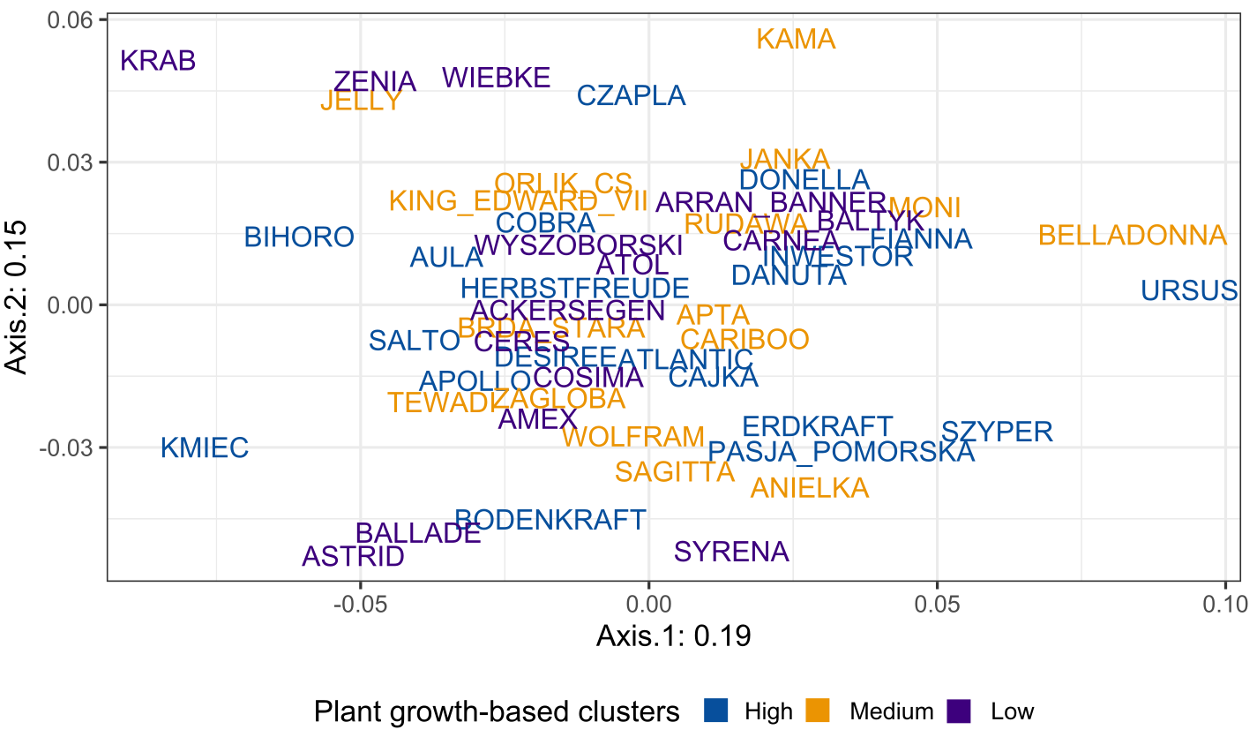


**Figure S7**. Ordination plots. Principal coordinate analysis (PCoA) of beta diversity in the rhizosphere fungal communities based on weighted UniFrac distance. Colors indicate the plant growth-based clusters to which each cultivar belongs (blue: high performing, orange: medium performing, purple: low performing).


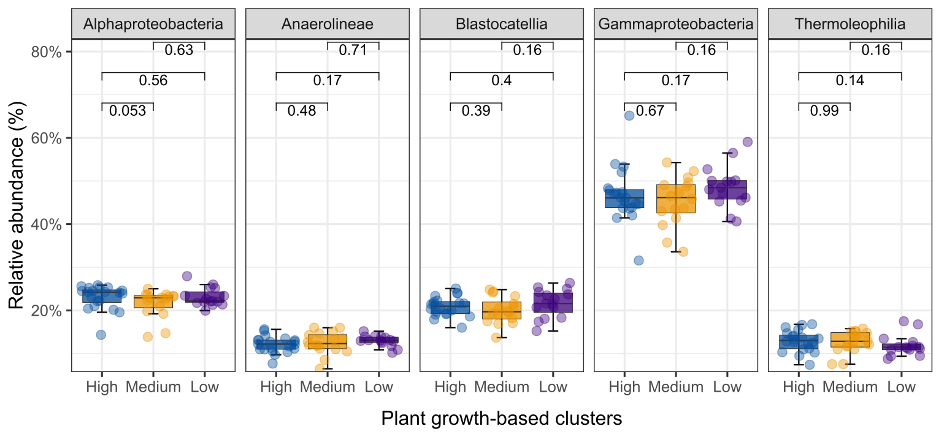


**Figure S8**. Analysis of bacterial community composition. Pairwise comparison of bacterial classes between the growth clusters. Boxplots display the medians, tops and bottoms of the boxes represent 75th and 25th quartiles, and whiskers outside this range; dots illustrate the individual observations in each cluster. Colors indicate the different potato clusters (blue: high performing, orange: medium performing, purple: low performing). Pairwise Wilcoxon test (*P* < 0.05) was applied to calculate significant differences across sample groups and numbers above the boxes indicate the corresponding *P*-values of the comparison.


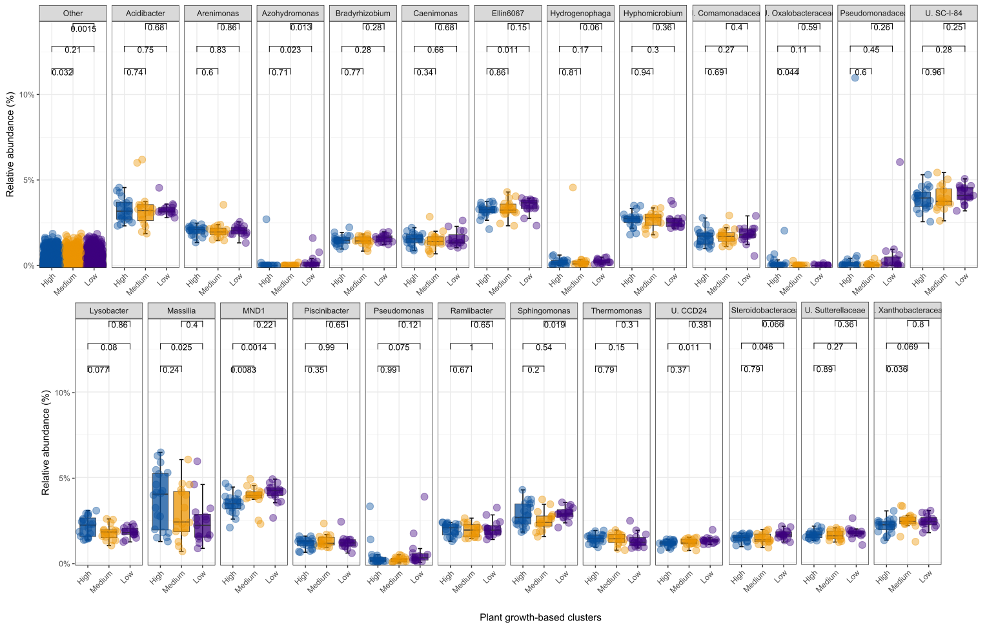


**Figure S9**. Analysis of Proteobacteria. Pairwise comparison of genera between the growth clusters. Boxplots display the medians, tops and bottoms of the boxes represent 75th and 25th quartiles, and whiskers the outsides of this range; dots illustrate the individual observations in each cluster. Colors indicate the different potato clusters (blue: high performing, orange: medium performing, purple: low performing). Pairwise Wilcoxon test (*P* < 0.05) was applied to calculate significant differences across sample groups and numbers above the boxes indicate the corresponding *P*-values of the comparison.


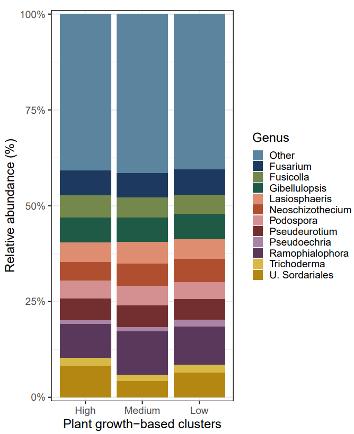


**Figure S10**. Analysis of fungal community composition. Relative abundance of the top 11 genera in the rhizosphere of the plant growth-based clusters of potato cultivars. Colors represent the individual taxa.


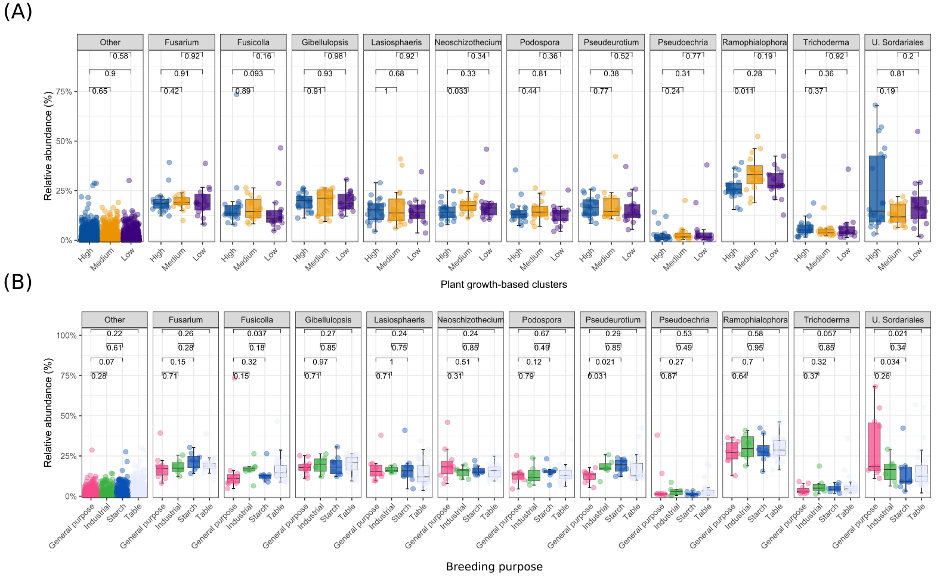


**Figure S11.** Analysis of fungal community composition. Pairwise comparison of fungal genera (A) between growth clusters and (B) breeding purpose. Boxplots display the medians, tops and bottoms of the boxes represent 75th and 25th quartiles, and whiskers outside this range; dots illustrate the individual observations in each cluster. Colors indicate the different potato clusters (blue: high performing, orange: medium performing, purple: low performing). Non-parametric Wilcoxon test (*P* < 0.05) was applied to calculate significant differences across sample groups and numbers above the boxes indicate the corresponding *P*-values.


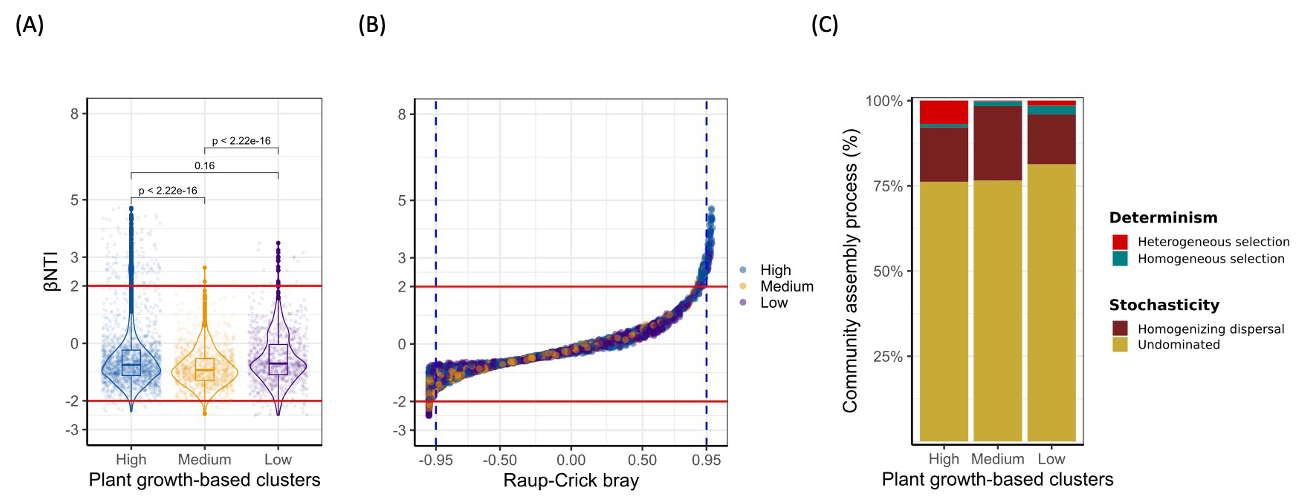


**Figure S12.** Community assembly processes dominating the rhizosphere fungal communities in response to the plant growth-based clusters of potato cultivars (A). Differences in beta-nearest taxon index (βNTI), together with Raup-Crick-based Bray-Curtis (B) served to determine the community assembly process that dominated the growth clusters (C). The thresholds for the |βNTI| = 2 and |RCbray| are highlighted as horizontal red lines and the vertical dashed blue lines, respectively. Differences in βNTI are based on Wilcoxon rank-sum test and were considered significant when *P*-value < 0.05. Colored bars represent the relative contribution of the different processes.


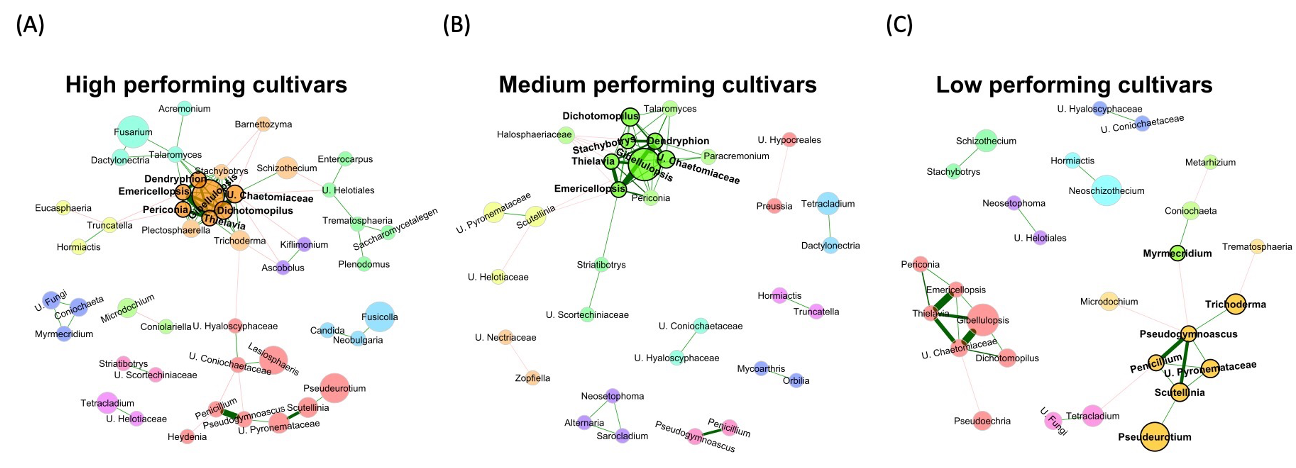


**Figure S13.** Fungal co-occurrence network analysis in the rhizosphere according to plant growth-based clusters of potato cultivars. Nodes indicate the genera and edges represent significant (p < 0.05) Pearson correlation between a pair of nodes. Edge colors display the nature of interactions (green: positive, red: negative). Node colors indicate the different modules. Nodes with thick black borders represent hub taxa whereas node sizes indicate their respective relative abundances.

# References

Taiz L, Zeiger E. *Plant Physiology*. 5th ed. Sunderland, MA: Sinauer Associates Inc, 2010.
